# Evolution of the batoidea pectoral fin skeleton: convergence, modularity, and integration driving disparity trends

**DOI:** 10.1007/s10682-025-10330-x

**Published:** 2025-02-17

**Authors:** Faviel A. López-Romero, Eduardo Villalobos-Segura, Julia Türtscher, Fidji Berio, Sebastian Stumpf, Richard P. Dearden, Jürgen Kriwet, Ernesto Maldonado

**Affiliations:** 1 EvoDevo Research Group, Unidad de Sistemas Arrecifales, Instituto de Ciencias del Mar y Limnología, Universidad Nacional Autónoma de México, Puerto Morelos, C.P. 77580 Quintana Roo México; 2 Evolutionary Morphology Research Group, Department of Palaeontology, Faculty of Earth Sciences, Geography and Astronomy, University of Vienna, Josef-Holaubek-Platz 2, 1190 Vienna, Austria; 3 Department of Zoology, Stockholm University, Svante Arrhenius väg 18B, 114 18 Stockholm, Sweden; 4 Vertebrate Evolution, Development, and Ecology, Naturalis Biodiversity Center, Darwinweg 2, 2333 CR Leiden, The Netherlands; 5 School of Geography, Earth and Environmental Sciences, University of Birmingham, Edgbaston, Birmingham, B15 2TT UK; 6 Vienna Doctoral School of Ecology and Evolution (VDSEE), University of Vienna, Djerassiplatz 1, 1030 Vienna, Austria

**Keywords:** Batoidea, Pectoral fin skeleton, Evolution, Modularity, Disparity, Convergence

## Abstract

Batoids (skates and rays) are the most speciose group of cartilaginous fishes with a diverse array of ecological adaptations and swimming modes. Early skeletal fossil remains and recent phylogenetic analyses suggest that convergence among batoids has occurred independently multiple times. The drivers for such disparity patterns and possible association with modularity and phenotypic integration among batoids are not fully understood. Here we employed geometric morphometrics and phylogenetic comparative methods to characterize the evolutionary trends in the basal fin skeleton of extinct and extant batoids and dorsoventrally flattened sharks. We found that the most speciose orders of batoids, Myliobatiformes and Rajiformes, display the lowest levels of morphological disparity, while Torpediniformes and Rhinopristitiformes have the highest disparity. Differences in evolutionary rates by habitat indicate that both reef and freshwater species evolved faster than deep-sea and shelf-distributed species. We further explored the differences based on swimming modes and found that species with oscillatory swimming exhibit higher evolutionary rates on their coracoid bar. We found that specific groups underwent different rates of evolution on each element of the pectoral fin. This was corroborated by the modularity and integration analyses, which indicate differences in the covariation between structures among the analyzed groups. The convergence analysis does not support the resemblance between flattened sharks and batoids; however we found convergence between extinct batoids and modern guitarfishes. Our findings suggest that habitat and swimming mode have shaped the pectoral fin evolution among batoids.

**Supplementary Information:**

The online version contains supplementary material available at 10.1007/s10682-025-10330-x.

## Introduction

**Fig. 1:**
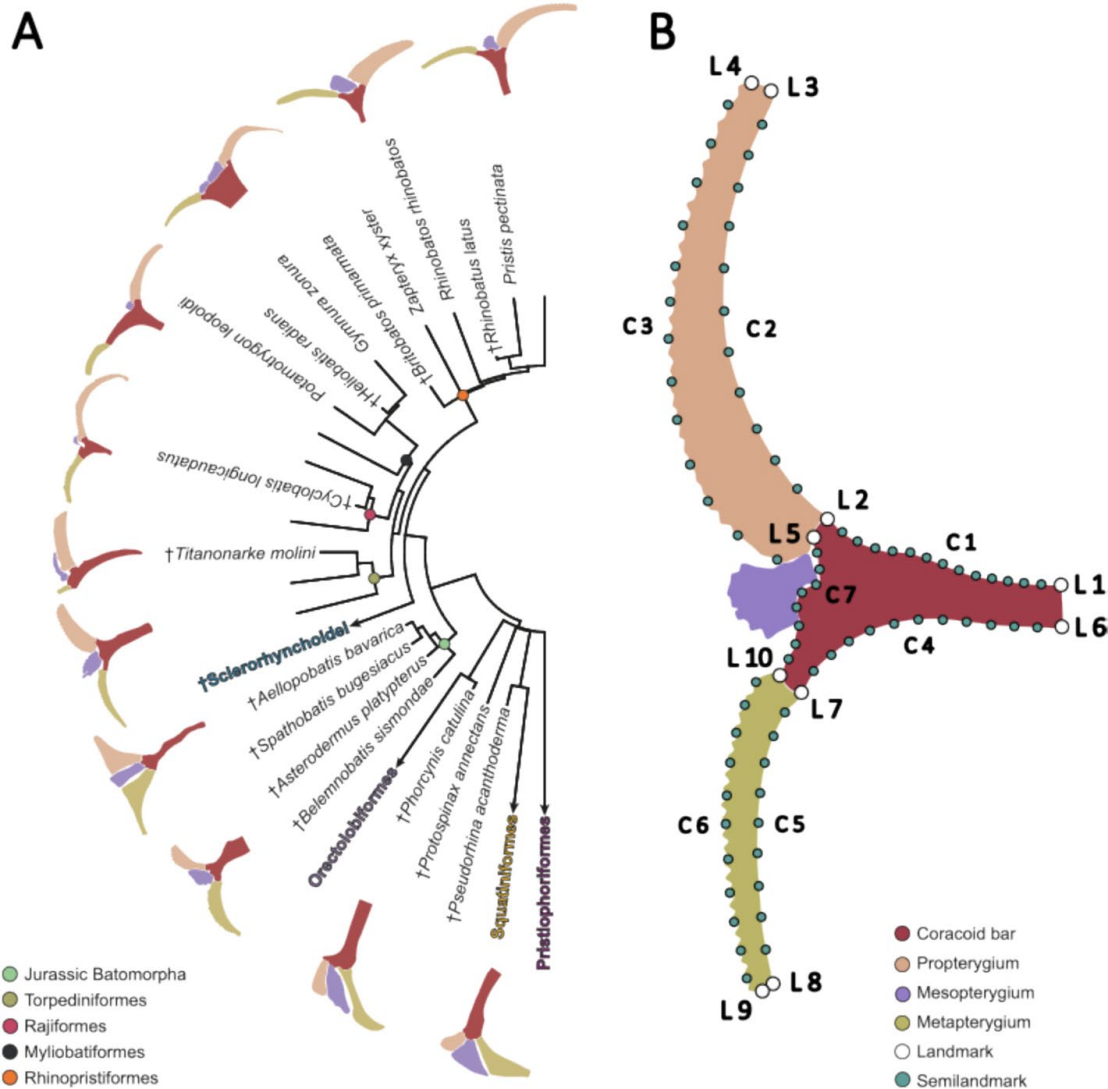
**A** Simplified phylogeny of the studied group of elasmobranchs displaying the corresponding skeletal anatomy of the pectoral fin. **B** Landmark coordinates scheme followed to perform the statistical shape analysis (L1-10 = fixed landmarks; C1-7 = Curve landmarks)

Rays, skates and guitarfishes (hereafter “batoids”) comprise the most speciose group of cartilaginous fishes, with nearly 620 species described to date (Weigmann 2016; Fricke et al. 2022). Batoids have diversified in several aquatic environments, from open ocean to freshwater and from nearshore reefs to the deep sea (Compagno 1990; Last et al. 2016). The most striking feature of batoids is their dorsoventrally flattened body, expanded pectoral fins to form a disc, which display a high diversity of shapes (Da Silva et al. 2018; Martinez et al. 2016; Franklin et al. 2014) (Fig. 1A). The basic structure of the pectoral fin is composed of three basal elements which articulate to the coracoid bar by condyles (Fig. 1A and B). Modifications of the pectoral fin skeleton like the expansion of the pectoral girdle in some myliobatiforms, and the number of radials supported by each basal element follow an evolutionary trend in relation with the swimming mode (Hall et al. 2018). The wing-like fins also show several modifications the radials showcase differences in the mineralization associated with the swimming type (Schaefer & Summers 2005). Batoids have a remarkably long fossil record with several groups represented by completely articulated specimens (Türtscher et al. 2024). The earliest remains of batoids are found in the Early Jurassic and several articulated specimens in the Middle Jurassic (Thiollière 1852; Underwood 2006; Stumpf & Kriwet 2019; Villalobos-Segura & Underwood 2020). Extinct representatives of modern groups of batoids showcase a wide morphological disparity in several traits which are interpreted as a mixture of plesiomorphic and derived features (Cappetta 1980; Kachacha et al. 2017; Marramà et al. 2018, 2021). Meanwhile, modern batoids diversified around the Lower Cretaceous, making them a long-standing group that has endured extinction events (Underwood 2006; Villalobos-Segura & Underwood 2020; Kriwet et al. 2009; Guinot et al 2012). This provides a unique opportunity to study the evolutionary trends associated to the pectoral fin across evolutionary time.

A dorso-ventrally flattened body has evolved independently in cartilaginous fishes, such as several Paleozoic forms, holocephalans, and Mesozoic forms to modern sharks (Lund 1988; Carvalho et al. 2008; Egeberg et al. 2014; Duffin et al. 2023). As the entire group, batoids are estimated to have evolved during the Permian after the split with their sister group the sharks (Renz et al. 2013). Batoids have acquired highly disparate body forms, like the sawfish with its elongated tooth-bearing rostrum to the bowmouth guitarfish with a large, muscular trunk that powers locomotion (Aschliman et al. 2012). Consequently, the resolution of the relationships within the main four orders (Rajiformes, Myliobatiformes, Torpediniformes and Rhinopristiformes) becomes relevant to assessing the patterns of morphological disparity. Even the relationships with their sister group (sharks) have presented different arrangements through the years (Compagno 1977; Shirai 1992; Carvalho 1996; Douady et al. 2003; Naylor et al. 2012, 2016; Amaral et al. 2018; Stein et al. 2018; Kousteni et al. 2021; Villalobos-Segura et al. 2022).

The outline shape of the pectoral fin seems to reflect some relation to their phylogenetic relationships. Previous studies indicate that highly specialized groups like stingrays display a high morphological disparity (Franklin et al. 2014; Martinez et al. 2016), which is explained by their swimming mode in terms of the aspect ratio of the fins (the area supported by plesodic radials) (Martinez et al. 2016). However, the internal skeletal features vary according to the ecomorphotypes, unlike the external shape (Hoffmann et al. 2020). This highlights the importance of assessing the anatomical features, to understand the underlying processes leading to convergence patterns. Because of batoids taxonomic and ecological diversity, their skeletal elements of the pectoral fin represent interesting characters to investigate how the configuration of the elements might relate to their body plan and swimming modes.

The extent to which distinct components of anatomical features are linked to each other, and the patterns of covariation they display are known as phenotypic integration (Klingenberg 2014). These patterns can indicate if two or more structures covary between them to consider them as a composed unit (module) (Klingenberg 2014). The extent of integration within a group has been associated to an increase or decrease in the amount of morphological disparity (Goswami et al. 2014). This is explained by the quasi-independent evolution of traits which follow different evolutionary trajectories compared to other traits, thus increasing disparity by the exploration of trait space (Felice et al. 2018; Zelditch & Goswami 2021). The variation is constrained, although this does not prevent the group from displaying differences in species diversity (Guillerme et al. 2020). Therefore, there can be groups with a high number of species but with low disparity, as well as low diversity with high disparity (Erwin 2007). Together, this can indicate trends through the history of taxonomic groups across time, such as the release of disparity following an event in the past and the related macroevolutionary consequences (Puttick et al. 2020). The patterns of modularity and integration are relevant because the traits can evolve in different trajectories, leading to specialized forms (Wagner et al. 2007). Among elasmobranchs, it has been shown that batoids display higher modularity signal in the skull than sharks, which might have facilitated their diversification driven by the prey-acquisition strategies (Gayford et al. 2024). However, little is known about the possible role of modularity and integration in the evolution of the pectoral fin, especially in relation to swimming.

Our study aims to understand the drivers of the pectoral fin shape evolution in a major group of cartilaginous fishes. First, we explore the morphological variation of the skeletal elements composing the base of the pectoral fin among extant and extinct batoids to determine patterns of disparity. We then investigate if individual elements of the pectoral fin or the three composite structures undergo different evolutionary rates in relation to swimming type and habitat and study whether morphological disparity of the structures represents a similar pattern as the evolutionary rates. Additionally, we investigate whether modularity or integration has contributed to determine the shape variation of the pectoral fins between the taxonomic orders of batoids, both extant and extinct, and between sharks and batoids. Finally, we evaluate the convergence among several groups of batoids and across specific groups of batoids and sharks, to elucidate possible shape resemblance due to similarities in swimming type and habitat.

## Material and methods

### Data acquisition

We gathered information of the internal anatomy of batoids with several radiographs taken from available depositories in iDigiBio. The depositories contain information from the following museums: Smithsonian Institution, National Museum of Natural History (USNM), Natural History Museum, London (NHMUK), Museum national d'Histoire naturelle (MNHN), Australian Museum (AM), California Academy of Sciences (CAS), Florida Museum of Natural History (FLMNH), Harvard University, Museum of Comparative Zoology (MCZ), Naturhistorisches Museum Wien (NMW), Field Museum of Natural History (FMNH). Segmentations from CT-Scans were performed with the software Slicer3D (v. 5.2.2), Mimics (v. 23.0) (Materialise), and screenshots of the 3D images were taken in ventral view. The CT-Scans are available from Kamminga et al. (2017), Morphosource (morphosource.org), and Chondrichthyan Tree of Life. Additionally, photos of fossil specimens come from museum collections: Swedish Museum of Natural History (NRM), Staatliches Museum für Naturkunde Stuttgart (STU), NHMUK, MNHN, AMNH). A total of 362 specimens from which 330 specimens belonging to batoids, representing 194 species, 32 specimens of sharks representing 17 species. We verified the taxonomic assignment in the Eschmeyer Catalog (Fricke et al. 2022) and FishBase (Froese & Pauly 2024). From FishBase, Aquamaps, and the literature we obtained information regarding depth distribution and environmental occurrences for each species. For fossil species, we obtained information regarding the environment with the Paleobiology Database (Uhen et al. 2023) and from literature.

### Phylogenetic reconstruction

We examined the phylogenetic relationships of the various elasmobranch taxa using a modified data matrix of Villalobos-Segura et al. (2022) to provide a phylogenetic context for the macroevolutionary analyses. The only modifications made to the matrix were in the number of terminals, to accommodate the increased number of taxa included in the current analysis. The matrix includes fossil chondrichthyans from the Paleozoic, Mesozoic, Cenozoic, and recent taxa. The fossil species †*Doliodus latispinosus* (Whiteaves, 1881) served to root the phylogenetic analysis. Two positive constraints were enforced, one for the whole batoids to ensure that no wild card taxa fell outside this group. This constraint was not necessary as the analysis can be carried out without it, since there were no batoid taxa falling out of this group. Another constraint was inflicted on the Torpediniformes, to include all molecular groupings at the order level and to accommodate the inclusion of the recent taxa without radically increasing the number of characters and performing an extensive anatomical study, which would be beyond the scope of the present study (see electronic supplementary material) (Naylor et al. 2012; Aschliman et al. 2012; Last et al. 2016). The remaining phylogenetic associations were left unconstrained, to ensure reflecting the phylogenetic uncertainty associated to the morphological characters and the discrepancy between the phylogenetic hypotheses under morphological and molecular data. The resulting data matrix was assembled in Mesquite (v. 3.81) (Maddison & Maddison 2019) and contains 253 terminals and 143 characters (see electronic supplementary material at https://github.com/Faviel-LR/Batoid_Fins/tree/main/PhyloBat).

A parsimony analysis was conducted using TNT (v. 1.6) (Goloboff & Morales 2023). A new technology search was performed with 1,000 ratchet iterations, TBR (tree bisection and reconnection) and SPR (subtree pruning and regrafting) were used as the algorithm for branch permutations, holding one tree, additionally ten cycles of Tree drifting. This search was performed until 10 hits of the minimum score tree was reached. This search protocol was run ten times, saving the trees found on each search. All ten searches recovered the same strict consensus (see electronic supplementary material), suggesting an adequate search of the tree space. All the most parsimonious trees recovered in these ten searches were kept, but only the trees with unique topology were later used in the macroevolutionary studies. Tree branch lengths and likelihood scores were calculated using PAUP (v. 4.0a) (Swofford 2003) under the Mkv model with the gamma rate parameter, following the approach used by Brazeau et al. (2020).

### Geometric morphometrics

A landmark configuration was used to describe the shape of the pectoral fin (Fig. 1B, supplementary Table 1). We considered the coracoid bar, the first element of the propterygium, and the first element of the metapterygium. The mesopterygium was excluded from the analysis since it is not always present, or it was fused with the radials. Only the first element of the propterygium and metapterygium, respectively, were used for comparison, because the number of elements was variable between species. Only the first element was consistently present, allowing the assumption that these elements are homologous across taxa. The 2D landmarks and semilandmarks coordinates were captured with TPSDig2 (v. 2.31) (Rohlf 2006). The coordinates were then subject to a Generalized Procrustes Analysis (GPA) using the bending energy to slide the semilandmarks (Rohlf & Slice 1990; Gunz & Mitteroecker 2013). This was performed with the gpagen function from the R package geomorph (v. 4.0.7) (Adams & Otárola‐Castillo 2013). Coordinates partitions of each element were also subjected to a GPA, because we intended to trace shape changes of each individual element. The aligned coordinates were then used to perform a principal component analysis to visualize the variation among the individuals and explore the shape changes. This was performed for the full configuration and each element separately with the gm.prcomp function in geomorph. The original coordinates were then averaged to the species level and used with the phylogenetic hypothesis including fossil species to perform a phylogenetic aligned component analysis (PACA) (Collyer & Adams 2021) which aligns phenotypic data to the phylogenetic signal.

### Morphological disparity

We estimated the disparity per group using the sum of variances with all landmark configurations. We used the package DispRity (v. 1.8) (Guillerme 2018) and divided the set into the different taxonomic groups displayed in Fig. 1 (Rajiformes, Rhinopristiformes, Myliobatiformes, Torpediniformes, Squatiniformes, Orectolobiformes, Pristiophoriformes, Jurassic Batoids, Cretaceous Rhinopristiformes, sclerorhynchids, Cretaceous Rajiformes, and Eocene Myliobatiformes). We also considered the disparity of other grouping factors. We used a modified classification by Martinez et al. (2021) to compare between “deep sea”, “shelf”, “reef”, and “freshwater” occurring species. We compared the assigned swimming types into the categories “undulatory”, “oscillatory” (mostly present in Gymnuridae and Myliobatidae, and Mobulidae), and “axial undulatory”, since the pectoral fin is also linked to a swimming type (Rosenberg 2001; Schaefer & Summers 2005). We used the calibrated phylogeny and obtained the ancestral states for the nodes in the PACA. Together with this matrix and the phylogenetically aligned components (PAC) that explain 99% of the variation, we performed a disparity through time analysis using the sum of variances to observe changes throughout the history of batoids and sharks that could be associated with past geological events.

### Phylogenetic comparative methods

We used a subset of 100 random trees from the 420 trees obtained to perform the phylogenetic comparative methods to account for phylogenetic uncertainty. We trimmed the phylogeny to contain only the used taxa. We calibrated the tree with information about the first appearance in the fossil record for each group and the fossils used. We used the scaleTree function in the RRphylo package (v. 2.8) (Castiglione et al. 2018). We used the categories of habitat occupancy and swimming type to estimate the discrete evolutionary rates. First, we compared the support of the fitting of the Equal Rates, Symmetric, and All Rates Different models using the AIC and logLikelihod to evaluate the support using the fitMk function in castor (v. 1.8) (Louca & Doebeli 2018). With the selected model, we used the sim.map function in phytools (v. 2.1–1) (Revell 2024) to trace the history of traits. We performed the mapping on the 100 random trees and used these trees for the following analysis. From the PACA, we selected the components explaining up to 99% of the variation, these components were then used with the mvgls function in mvMorph (v. 1.1.9) (Clavel et al. 2015) to obtain the morphological evolutionary rates of the shape variables conditioned to the discrete traits as σ^2^. Additionally, we used these selected components with the mvgls, followed by the manova.gls function to estimate the association of shape with the categorical variables using Pillai’s test for significance. We performed this analysis on a set with only extant batoids.

We also used another approach to estimate the rates independently of discrete categories. We selected the phylogeny with the best score and calibrated the tree with information from the literature. With the calibrated tree we used the RRphylo function from the RRphylo package (v. 2.8) (Castiglione et al. 2018) to perform a phylogenetic ridge regression and obtain the rates per branch. With the search.shift function we compared the rates of the nodes of each group of interest (the main orders of batoids) to compare if each different taxonomic group has experienced a shift in the rates as regressions coefficients from the ridge arch regression (Castiglione et al. 2018). We also estimated the shift rates for each clade with the RRphylo search.shift function and verified the results consistency using 100 random trees with the overfitRR function. Because several instances of convergence have been suggested between groups of sharks and batoids and within groups of batoids, we performed a convergence test in RRphylo with search.conv (Castiglione et al. 2019). We took the Procrustes distances of the aligned coordinates and performed a cluster analysis using the UPGMA method. We then used this distance dendrogram with the phylogeny with the function cophylo from phytools to visualize the groups which converge into a cluster (Supplementary Fig. 1). We compared the resulting tangled groups to test for convergence. We performed other comparisons specific to groups like sclerorhynchids, Squatiniformes, Pristiophoriformes, and Pristidae. We performed a specific node search for convergence with these groups as well as between selachians and batoids to further explore a possible convergence often mentioned between angel sharks and batoids.

### Modularity and integration

Finally, the coordinate configurations were subdivided into five possible module hypotheses (Supplementary Fig. 2) considering: each element independent from each other (H1), a configuration with the propterygium and pectoral girdle as a module (H2), a configuration of the coracoid bar and metapterygium as a module (H2), a configuration of propterygium and metapterygium as a module separated from the coracoid bar (H3), and a null hypothesis of no modularity (H0). We compared the possible hypotheses with the compare.CR function in geomorph (Adams & Collyer 2019), which considers the covariance and the effect size to determine the strength of a modularity signal. The selected hypothesis, based on the covariance ratio effect sizes (Zcr), was used to compare the modularity signal by groups. We divided the subsets into different taxonomic orders of extant batoids, another comparison between extant and extinct batoids and a comparison of batoids and sharks. We estimated integration with two alternative methods. First, we used the partial least squares approach (Adams & Collyer 2016) with the defined modularity hypothesis from the previous analysis. We used another approach (Bookstein 2015) to assess the pattern of local or global integration changes in terms of self-similarity (no interpretable structure at any temporal scale). We compared the same groups as the ones for the modularity test.

**Fig. 2:**
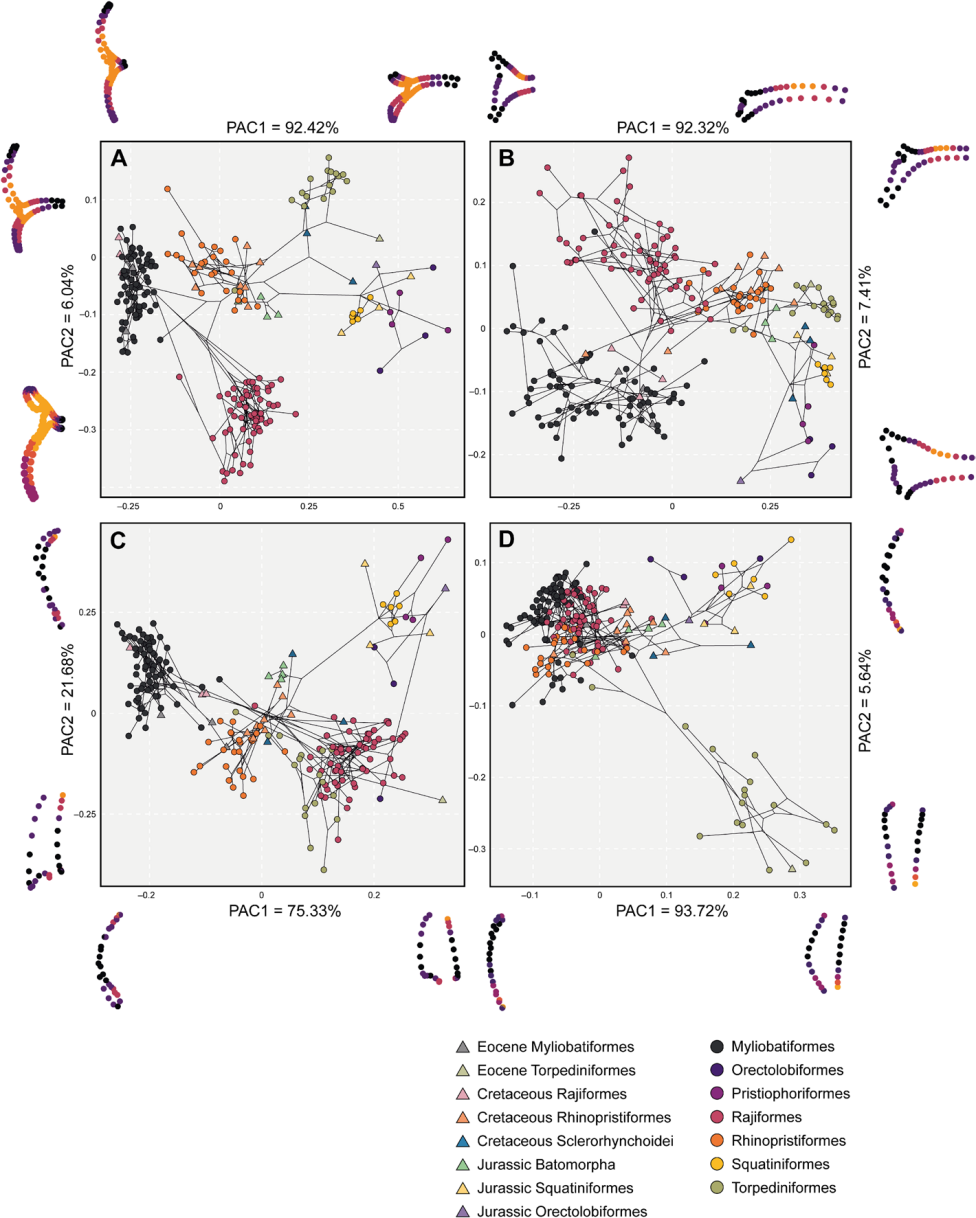
Phylomorphospaces for each landmark configuration, displaying averaged specimens at species level. Symbols on the right indicate the groups of each taxonomic order as circles and extinct groups as triangles. On each side of each axis, we show the extreme shapes for each component. **A** Full landmark configuration; **B** Coracoid bar configuration; **C** Propterygium configuration, and **D** Metapterygium configuration

## Results

### Pectoral fin shape variation

The results from the Principal Component Analysis (Supplementary Figs. 3–6) are similar to the ones derived from the phylogenetic aligned component analysis (PACA). The PACA shows that in the first component (PAC1) (92.42% of the variation), shape changes in the negative scores are associated with elongation of both propterygium and metapterygium, a short coracoid bar along the lateral axis and elongated in the joints with the propterygium and metapterygium (Fig. 2A). Most of the observed species in these scores correspond to Myliobatiformes and the extinct Rajiformes genus †*Cyclobatis* spp. In the positive scores, the coracoid bar extends laterally from the midline to the propterygium and metapterygium articulations, becoming slender. The sharks (Orectolobidae, Squatiniformes, and Pristiophoriformes) are located on these positive scores, without any overlap from a batoid species. Only a sclerorhynchid (†*Ptychotrygon rostrispatula* (Villalobos-Segura 2021) and a fossil torpediniform (†*Titanonarke mollini* (Jaekel, 1894)) seem to display a similar shape. Most of the species placed towards the mean values correspond to Rhinopristiformes, both extinct and extant. Jurassic batoids overlap Rhinopristiformes in the morphospace. Regarding PAC2 (6.04% of the variation), the minimum values show a medially short and posteriorly elongated coracoid bar where the metapterygium articulates. The propterygium is notably reduced in these scores while the metapterygium is quite elongated. All the species found here correspond to modern Rajiformes. On the positive PAC2 scores, the shape changes correspond to a medially elongated coracoid bar and a relatively short distance on the articular condyles, resembling the sharks' coracoid bar. The propterygium is elongated, however, it is not quite as long as in Myliobatiformes, whereas the metapterygium is shorter than the rest of the other ones in the morphospace. Overall, the positive PAC2 scores correspond to Torpediniformes (electric rays), which tend to display unfused coracoid bar on both antimeres and a notable reduction of the metapterygium. All the main orders of batoids are separated rather consistently in the morphospace when we consider the three structures at the same time in the analysis.

When each anatomical structure is analyzed separately, we observe a different pattern. The coracoid bar still represents a rather good trait for distinction and is consistent with the main groups (Fig. 2B). The PAC1 (92.32% of the variation) is explained by lateral elongation of the bar on the positive scores and narrow distance on the articulation region. In the negative scores, the coracoid bar is short and wide (antero-posteriorly) in the articulation area. Myliobatiformes and Rajiformes are distributed in a gradient-like pattern along these scores, followed by Rhinopristoformes and at the extreme the Torpediniformes and finally sharks in the positive scores. The second PAC2 (7.41% of the variation) explains mostly the elongated posterior articulation region of the coracoid bar, where the metapterygium attaches and a slender bar, Rajiformes are in these scores. The negative scores show a wide coracoid bar, especially in the anterior part as seen in most members of Myliobatiformes like Gymnuridae and Rhinobatidae. The propterygium PACA (Fig. 2C) displays a relatively good separation among groups. The propterygium is short on the negative scores of the PAC1 (75.33% of the variation) with a wide articular facet, strongly curved inwards with an anterior elongation which tapers. The shapes found on the positive scores correspond to sharks which have an overall short propterygium. These are followed towards the negative score by the Rajiformes and Torpediniformes, which overlap in this part of the morphospace. Meanwhile, the Jurassic batoids, sclerorhynchids, and Rhinopristiformes overlap in the middle. Myliobatiformes are located on the negative extreme values. The PAC2 (21.68% of the variation) on the positive scores shows a rather bulky anterior portion of the propterygium that tapers in the articular region. In the negative scores, the propterygium becomes more rectangular in shape with a narrow articular region. The extreme positive PAC2 shape is not usually seen among any group, although it can be similar in some fossil shark forms like extinct †*Pseudorhina* spp. although this one has a wider articulation region. The shapes in the negative scores correspond to members of the Torpediniformes. Finally, the metapterygium (Fig. 2D) shows a rather unique pattern with an overlap of Rajiformes, Myliobatiformes, and Rhinopristiformes, along with fossil forms. The PAC1 (93.72% of the variation) explains changes in the posterior elongation of the metapterygium. The positive scores display a rather short and curved metapterygium, while the metapterygium is slender and posteriorly elongated in the negative scores. Only Torpediniformes display a short metapterygium and this is indicated as a divergence in shape from the main cluster. The PAC2 (5.64%) shows a similar pattern with the positive scores expressing an elongated and curved metapterygium, while the negative scores typify a short and straight on the outer side metapterygium.

### Phylogenetic signal

Our results suggest that the strongest separation between groups occurs when all structures are considered. However, both the coracoid bar and propterygium show a consistent separation between groups with some overlap. This suggests that the phylogenetic signal also varies for each structure conversely to analyzing all three structures together. Indeed, the phylogenetic signal for the whole configuration indicates that there is a signal mostly separating clades (λ = 0.9696; Kmult = 1.5387), while this signal is reduced with more internal variation within the clades when the structures are analyzed separately. In this regard, the coracoid bar still displays a high phylogenetic signal (λ = 0.8564; Kmult = 0.7864), followed by the metapterygium (λ = 0.9223; Kmult = 0.7585) and propterygium (λ = 0.8752; Kmult = 0.6262).

### Morphological disparity by groups and through time

**Fig. 3:**
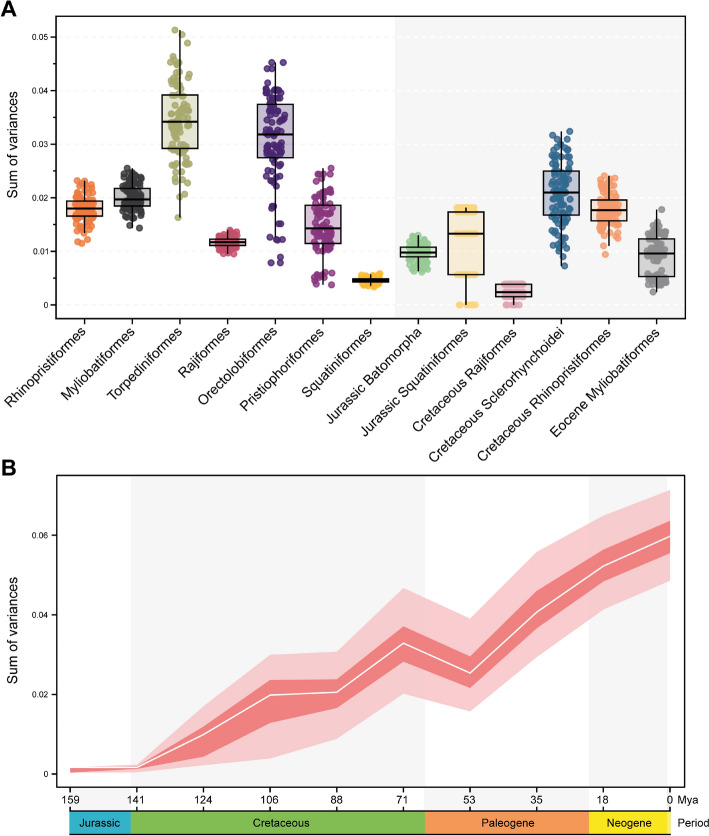
Morphological disparity estimated as the sum of variance over 100 bootstraps by **A** taxonomic groups; **B** through time

**Fig. 4:**
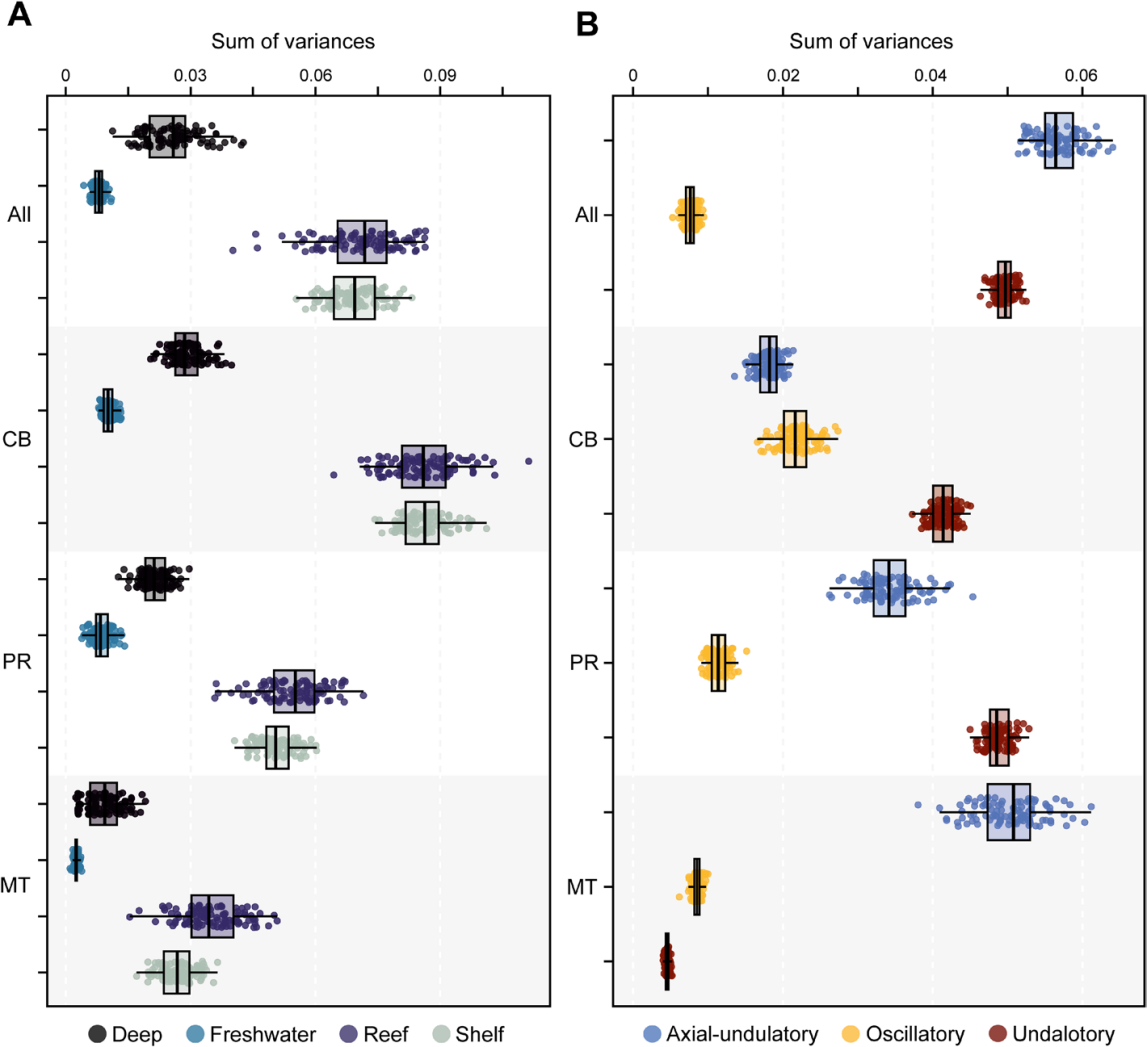
Morphological disparity estimated as the sum of variance over 100 bootstraps by A, habitat groups; B, and by swimming type. All: All elements; CB: Coracoid Bar; PR: Propterygium; MT: Metapterygium

We estimated the disparity of the different groups to discern whether there are differences between extant and extinct counterparts, as well as different habitat groups and swimming types. When considering the full configuration of landmarks, the estimated Procrustes variance for the morphological disparity shows that Torpediniformes display the highest disparity among all the groups. They are followed by Orectolobiformes, although they are represented by only few specimens and do not show the full disparity extent among Orectolobiformes. Squatiniformes displays the lowest disparity among the extant groups (Fig. 3A). Interestingly, despite being the most speciose, both Myliobatiformes and Rajiformes display low disparities (Fig. 3A). The extinct sclerorhynchids have the highest disparity among them all, followed by Cretaceous Rhinopristiformes. The Jurassic batoids, conversely, display low disparity. Overall, the extinct taxa do not display higher disparity than extant groups, except for the sclerorhynchids in either the full configuration or when isolated elements are considered (Supplementary Table 2). In view of this pattern, we explored a temporal component to reveal the disparity through time. For both the complete set and the set with batoids, there only is a pattern of gradual increase in disparity (Fig. 3B; Supplementary Fig. 7) starting in the Jurassic when the first holomorphic batoids appear in the fossil record. There is a steady increase in disparity until the Cretaceous when it reaches a maximum, followed by a sudden decline during the KPg extinction event. This reduction of disparity is followed by a constant increase from the Eocene persisting until today, suggesting a recovery delay of disparity right after the KPg extinction event. The disparity through time for each landmark configuration shows a very similar pattern as the one shown for the whole landmark configuration (Supplementary Fig. 8). The disparity of habitat occupancy shows the highest disparity occurs among the reef and shelf associated species (Fig. 4A and Supp. Table 2). Deep-sea species display an overall low disparity; most species in this category are rajiforms, which already display low disparities. Finally, the freshwater groups display the lowest disparity among all the groups. This category includes only members of the Potamotrygonidae (Myliobatiformes), which are highly specialized batoids in respect to their lifestyle. The comparison of each individual element and the whole configuration display very similar results for every habitat category (Fig. 4A, Supplementary Table 2). Regarding the swimming type, we found that species using axial-undulatory display the highest disparity, followed by the undulatory, and finally oscillatory type (Fig. 4B). In the case of the oscillatory swimming type, this might be expected because only families of Myliobatiformes are in this category (Aetobatidae, Gymnuridae, Myliobatidae, and Mobulidae) (Supplementary Table 2). However, analyzing the elements separately, we observe that the disparity of the coracoid bar in the undulatory swimming species is higher than the other two categories. Disparity of the propterygium is higher among undulatory swimming species, followed by axial undulatory species. Finally, the disparity of the metapterygium indicates that the axial undulatory type is higher than the other two categories (Supplementary Table 2). Considering the pairwise comparisons for all examined groups, we found that for most of the cases a clear difference in their disparity regardless of the subdivision of the compared landmarks or as a whole configuration including all the elements (Supplementary Tables 3–6).

### Evolutionary rates

**Fig. 5:**
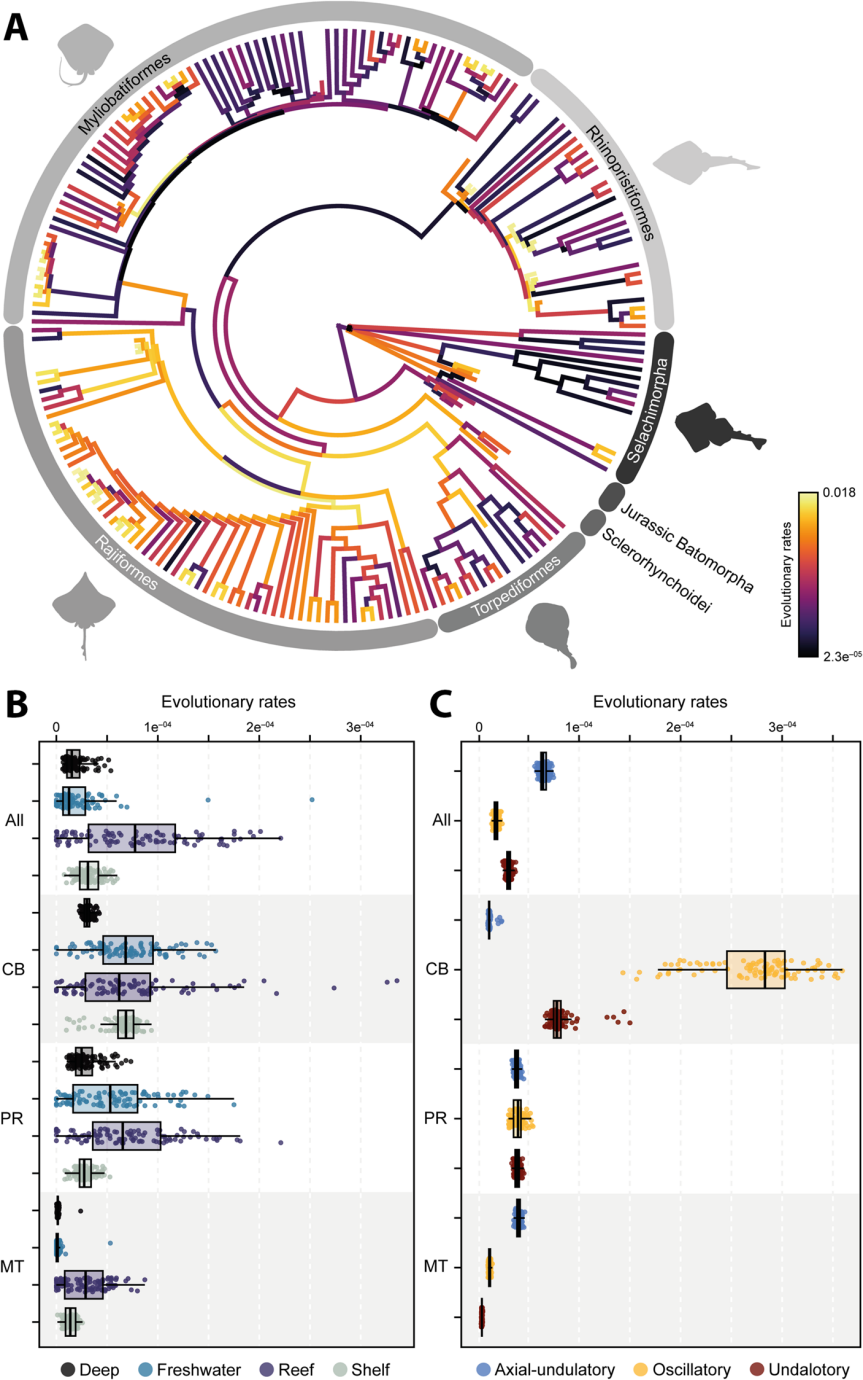
Morphological evolutionary rates of each of the 100 trees between **A** each taxonomic group; **B** Habitat; **C** and Swimming type. The values on the tree are expressed as absolute rates from the tips after ridge arch regression. The rates for the two remaining groups are expressed as sigma σ^2^. All: All elements; CB: Coracoid Bar; PR: Propterygium; MT: Metapterygium

Subsequently, we investigated whether the morphological evolutionary rates display a shift in the phylogeny when considering the shape variables. The results indicate a shift in rates in only a few batoid orders (Fig. 5, Supplementary Table 7). The sclerorhynchids are among the extinct taxa that display a positive shift in rates, and among extant taxa, there seems to be a positive shift in Rajiformes, whereas Myliobatiformes display a significant negative shift when the full configuration is considered. In the case of the coracoid bar, we find that almost all batoid clades experienced a positive shift, while selachians do not appear to display a significant shift (Supplementary Figs. 9, 10 and 11). The rates for both Myliobatiformes and Rhinopristiformes appear to be higher than for the other orders. For the propterygium, we found that only Jurassic batoids and selachians do not display a significant shift in the evolutionary rates. However, there is a significant shift at the basal node of all batoids. Similarly, the metapterygium displays a pattern of positive shifts in the examined main clades, while selachians do not display a significant shift at all. All these results present a consistent pattern when accounting for phylogenetic uncertainty (Supplementary Tables 7 and 8), with 100% of all the instances finding the same shift for the analyzed clades.

The evolutionary rates expressed as σ^2^ for the swimming type and habitat occupancy performed on the extant set, indicates that for the whole landmark configuration, shelf species tend to evolve faster, although the effect is not significant (Supplementary Tables 9–10). The coracoid bar shows a significant effect, with the reef species displaying high evolutionary rates, followed by freshwater species (Fig. 5B). This is similar to the results for the propterygium, which shows a statistically significant effect (Supplementary Tables 9–10). In the case of the metapterygium, the habitat does not appear to have a significant effect on the shape, although reef and shelf species display higher rates than deep sea and freshwater species. Regarding the swimming type, we find that, considering the whole configuration, the axial undulatory type evolved faster than the undulatory and oscillatory types (Fig. 5C and Supplementary Tables 9–10). However, for the coracoid bar, the oscillatory type has the highest rates than the two other types. This is also the case for the propterygium rates. In all these comparisons for swimming types, a significant effect on the shape variables is discernible, except for the metapterygium configuration.

### Morphological convergence

We next sought to investigate a possible pattern of evolutionary convergence among Torpediniformes, Rhinopristiformes, sclerorhynchids and Jurassic batoids, because of their high overlap in the morphospace. We compared the clustering results of the tanglegram of the Procrustes distances dendrogram and the calibrated phylogeny. We identified a cluster that converges towards the shape of Jurassic Batoids and Rhinopristiformes (Supplementary Fig. 1). Another group formed by †*Cyclobatis* spp. plus Myliobatiformes, and a group consisting of sclerorhynchids, Pristidae, Squatiniformes, and Pristiophoriformes. Therefore, we explored convergence patterns among these groups. Overall, the results indicate that there is no convergence in the shape trajectories between sharks and batoids when comparing both clades’ nodes (Table 1). We only found evidence of convergence when comparing the node of †*Cyclobatis* spp. which converges to the node of †*Asterotrygon maloneyi* (Carvalho et al. 2004) and †*Heliobatis radians* (Marsh 1877). Moreover, the results from convergence using the cluster groups suggest convergent trajectories between extant Rhinopristiformes and Jurassic batoids, and between extant and extinct guitarfish-like species. We found no convergence between extinct Rhinopristiformes and Jurassic batoids (Table 1). Likewise, convergence was found between members of Sclerorhynchidae, Pristidae, and Pristiophoriformes, and between Squatiniformes and Sclerorhynchidae (Table 1).

**Table 1:** Results from the convergence tests on the state condition comparing groups

State 1	State 2	Angle state	Angle state time	p angle state	p angle state time
E_Guit	F_Guit	27.438	0.188	0.002	0.016
E_Guit	Jbatoid	60.719	0.224	0.02	0.043
F_Guit	Jbatoid	49.452	0.276	0.018	0.332
Pristidae	Scle	43.406	0.164	0.017	0.021
Pristidae	Ppho	58.099	0.103	0.055	0.002
Scle	Ppho	30.881	0.065	0.006	0.001
Scle	Ange	30.969	0.073	0.001	0.001
Node 1	Node 2	Angle by clades and ancestors	*P* value		
*Cylcobatis*	*Heliobatis* + *Asterotrygon*	0.0498308	0.005		

(State1 and State2) and indicating the significance of the shared angle trajectories between the compared groups. Significant results are in bold at a < 0.05 threshold

### Modularity and integration

The modularity analyses suggest that there is strong support for a separation of the elements as individual modules, i.e., each skeletal element covaries independently (Supplementary Table 11). Although the configuration considering the propterygium and metapterygium as a single module shows some support (Supplementary Table 11), the effect size indicates that the separation of each element is better supported. By comparing the configuration between the groups of batoids, we found that both members of Myliobatiformes and Rajiformes present a higher modularity signal compared to those of Rhinopristiformes and Torpediniformes (Table 2). When we compared the modularity signal of the extant batoids, we found that members of both Rajiformes and Rhinopristiformes appear to display a higher signal than the other two orders, and the signal is significant (Table 2). The comparison between extant and extinct batoids suggests that fossil taxa have a higher modularity signal, although the effect is not significant (Table 2). When comparing batoids and dorsoventrally flattened sharks, we found that the batoids have a significantly higher modularity signal. The integration strength signal for the groups described above, shows that among extant batoid orders, members of Rajiformes and Myliobatiformes are more integrated than the other two extant orders (Table 3). Extant batoids appear to display a much higher integration than fossil forms. Finally, the comparison between batoids and dorsoventrally flattened sharks indicates that batoids present a more integrated phenotype in the pectoral fin than sharks (Table 3). All these results are consistent with the findings from the global integration test for all comparisons we made (Table 3).

**Table 2:** Results from the modularity tests between each group

(a)						
	Rajiformes	Myliobatiformes	Rhinopristiformes	Torpediniformes		
CR	0.891	0.794	0.912	0.822		
ES	− 9.233	− 8.956	− 9.269	− 7.344		
(b)						
Rajiformes	–	**0.020**	0.618	**0.014**		
Myliobatiformes	2.31	–	**0.005**	0.606		
Rhinopristiformes	0.497	2.75	−	**0.005**		
Torpediniformes	2.435	0.515	2.779	–		
(c)						
	Extant Batoids	Fossil Batoids			Batoids	Sharks
CR	0.823	0.895			0.848	0.92
ES	− 8.521	− 6.808			− 8.506	− 6.222
(d)						
Extant Batoids	-	0.198		Batoids	–	**0.042**
Fossil Batoids	1.285	–		Sharks	2.026	–

(a) Extant batoid orders CR and ES value; (b) Pairwise comparison of the modularity ES value (lower triangle) and p value (upper triangle). (c) CR and ES value of extant batoids vs fossil batoids, and batoids vs sharks. (d) Pairwise comparison between Extant batoids vs fossil batoids, and batoids vs sharks CR = covariance ratio. ES = Effect size. Significant results are in bold at a < 0.05 threshold

**Table 3:** Results from the integration test between each group

‍(a)						
	Rajiformes	Myliobatiformes	Torpediniformes	Rhinopristiformes		
ES	5.717	5.696	3.224	4.37		
R-pls	0.903	0.862	0.88	0.951		
GI	− 0.998	− 1.103	− 1.061	− 1.015		
(b)						
Rajiformes	–	0.686	**0.002**	0.248		
Myliobatiformes	0.403	–	**0.005**	0.427		
Torpediniformes	3.033	2.751	–	0.076		
Rhinopristiformes	1.153	0.794	1.771	–		
(c)						
	Extant Batoids	Fossil Batoids			Batoids	Sharks
ES	7.581	3.524			6.771	2.678
R-pls	0.899	0.932			0.917	0.947
GI	− 1.182	− 1.016			− 1.154	-0.915
(d)						
Extant Batoids	–	**0.00002**		Batoids	–	**0.00002**
Fossil Batoids	4.185	–		Sharks	4.2	–

(a) Extant batoids orders values for ES, R-pls, and GI. (b) Pairwise comparison of the ES values between extant orders (lower triangle) and associated p value (upper triangle). (c) ES, R-pls, and GI values of Extant batoids vs fossil batoids, and batoids vs sharks (d) Pairwise comparison between Extant batoids vs fossil batoids, and batoids vs sharks (ES values in the lower triangle and p values in the upper triangle)ES = Effect size. R-pls = r partial least squares value, GI = global integration value. Significant results are in bold at a < 0.05 threshold

## Discussion

A fundamental aspect of evolutionary biology is to understand the drivers that have led to the evolution of novel phenotypes. As a means for movement, the fins in aquatic organisms represent a structure that might be under several selective pressures and thus respond differently in each individual clade which resulted in convergences in different groups (Donley et al. 2004; Fish 2023). This results in differently adopted strategies, which ultimately have an impact on their patterns of morphological disparity and evolution. Batoids display a wide array of habitat distribution and swimming types, from the deep-sea/walking skates, to the pelagic/”flying-like” manta rays. It is necessary to understand the impact of such diverse strategies and habitats, as well as processes like modularity and integration across time, in shaping the evolutionary history of their pectoral fin. We approached this by sampling modern and fossil species encompassing major groups, habitats, and swimming types. The pectoral fins, specifically the basal elements, represent a trait with a primary function in movement through the environment. Because of the modular nature of its components, it can inform about specific constraints which have an impact on their disparity and convergence patterns. From our results we found that the pectoral fin among batoids has undergone specific changes for each clade and basal element throughout their history. Despite being the most speciose groups, Rajiformes and Myliobatiformes have lower disparities and in the case of Myliobatiformes, lower evolutionary rates.

Previous findings from the fin outline shape suggest that members of Myliobatiformes have attained higher disparity (Franklin et al. 2014; Martinez et al. 2016; Marramà et al. 2023). Interestingly, we found that this pattern is not necessarily the same for the basal fin elements. This could indicate that the higher integration also observed in such groups can allow other parts like the radials to vary independently and achieve higher disparity. From their appearance in the fossil record, the disparity of basal elements of the pectoral fin in batoids constantly increased. Novel phenotypes found during the Eocene from Monte Bolca (Marramà et al. 2021, 2019), for instance, demonstrate that batoids experienced a delayed recovery after the KPg massive extinction. This is also matched by observations of the diversity through time of the fossil record (Guinot et al. 2012; Guinot & Cavin 2020). Among sharks, recent research shows that high disparity was achieved in the Late Cretaceous (Sternes et al. 2024), which is coincident with our findings of disparity through time. The evolution of novel phenotypes resulting in an increased diversity could be associated with the increase of continental fragmentation observed during the Jurassic and Cretaceous, eventually leading to more nearshore environments (Guinot & Cavin 2020). This could have led groups like Rajiformes, considered for a long time as an invariant group (McEachran & Dunn 1998), to diverge towards different phenotypes, such as for instance †*Cyclobatis* spp. with its morphology resembling the one observed among freshwater Myliobatiformes (Forey et al. 2003). Other contemporary representatives of the group display a more “rajiform shape” (e.g., †*Raja davisi* (Fowler 1958)). These fossils illustrate a wider range of disparity in the past, compared to modern groups. In the case of Rajiformes, these changes can also be seen in their present distribution with only a few members in tropical or subtropical waters, but mostly distributed in either temperate to boreal zones or deep sea (Ebert & Compagno 2007). On the other hand, a consistent pattern of the body shape is discernible among the sharks analysed in this work such as Squatiniformes since their origin according to the fossil record (Carvalho et al. 2008). Interestingly, the highest disparity attained in reef and shelf habitats differs substantially from other groups like bony fishes, which tend to display a shifted pattern of disparity diversity (Martinez et al. 2021). Probably the absence of Rajiformes in reefs, along with the complex array of species in these regions yields a higher disparity among reef Batoids.

We found that the early diverging members of each main order tend to display a generalized morphology, similar to the one observed among guitarfishes. This is supported by the convergence tests we conducted (Table 1). A convergent trajectory was not found between sharks and batoids, only between sharks and the extinct group of sclerorhynchids (Table 1). Recent developmental studies suggest that the anterior expansion of the pectoral fin is under the control of the *Shh* signalling pathway (Dahn et al. 2007) and that these changes are associated with chromosome architecture during development (Marlétaz et al. 2023). In addition, Hox genes expression patterns observed in other groups like bamboo sharks, show that the retinoic acid signalling contributes to the posteriorization of the pectoral fin (Onimaru et al. 2015). This signalling alteration leads to a shark-like fin phenotype in skates (Nakamura et al. 2015). Nevertheless, the pectoral fin diversity we show suggests that the modification of the pectoral fin also involves the expansion of single elements like the propterygium and metapterygium. This expansion is lacking among sharks, although up to date no developmental studies have been conducted on either saw sharks or angel sharks to confirm such an interpretation. However, the description of developmental sequences in these groups suggests that fin expansion occurs earlier among batoids than in angel sharks (Natanson & Cailliet 1986; Maxwell et al. 2008).

From our analyses, the strength of the phylogenetic signal observed differs when different parts of the pectoral fin were analysed, as well as the evolutionary rates for each structure. As in other vertebrates with highly divergent morphologies (Felice & Goswami 2018; Bardua et al. 2020; Coombs et al. 2022; Larouche et al. 2023; Law et al. 2024), changes in batoids may follow a mosaic evolution. In addition to the pectoral fins, other features that are unique for the group like the fusion of the most anterior vertebrae (synarcual), the uniquely found anteorbital cartilage, and the dorsally fused suprascapulae, suggest that several changes occurred during the evolution of the group that led to the present morphologies. Some of the Cretaceous and Eocene taxa belonging to extant groups display morphologies with a mixture of characters between different orders (Marramà et al. 2019). More recently, the so-called aquilopelagic phenotype has been shown to have had evolved together with many other modifications for durophagy (Marramà et al. 2023). The radial elements of the pectoral fin also evolved differently than the basal supporting skeletal elements of the fin depending on the swimming type of each species (Rosenberg 2001; Hall et al. 2018). Nevertheless, some genera like *Gymnura* show that the swimming speed is relevant for displaying a specific swimming type (Kim et al. 2017). Our results indicate that higher evolutionary rates in the coracoid bar shape present a trend for the groups that display oscillatory swimming type. Previous works on the pectoral fin shape support a strong phylogenetic signal among batoids which can predict the taxonomic group and the swimming type (as defined by the aspect ratio value of the fin) (Franklin et al. 2014). Although some further refinement corroborated this observation, following a landmark scheme that better represented the homology between compared fins (Martinez et al 2016). These findings indicate that the shape changes in batoids occur along the axes of maximum variation and correlated with the aspect ratio (Martinez et al. 2016). Furthermore, this axis of shape change variation of the fin in relation with the swimming type suggests that the evolution of novel phenotypes in batoids facilitated the transition from benthic to pelagic forms (Marramà et al. 2023).

It has been suggested that extreme morphologies are achieved due to a higher phenotypic integration, which also can be related to low disparity (Goswami et al. 2014; Felice et al. 2018; Guillerme et al. 2020). Our results from the integration test indicate that members of both Rajiformes and Myliobatiformes present the highest integration signal among extant batoids. Interestingly, extant batoids appear to have a higher integration than extinct ones. This pattern also was observed in other structures like the skull in angel sharks (López-Romero et al. 2020), where extinct forms tend to display lower levels of integration and higher morphological disparity. We found that extinct sclerorhynchids, which attained extreme morphologies resembling the ones seen among sharks (Wueringer et al. 2009; Villalobos-Segura et al. 2021), display higher disparity than some modern batoids. In the case of Torpediniformes, where the disparity is higher compared to the rest, they represent a group with several forms resembling Rhinipristiformes (*Platyrhina* and *Plathyrinoidis*), which can contribute to their increased disparity. From the modularity analysis we observe that Torpediniformes have a higher signal compared to Rajiformes and Rhinopristiformes, which can explain the increased disparity. By comparing batoids and sharks, the pectoral fin of flattened sharks displays a less integrated phenotype and higher modularity. This would suggest that the processes involved in the evolution of the dorsoventral flattening of sharks might not be similar to the ones experienced by batoids. The results of modularity and integration suggest that both signals are significant for the examined groups. However, it has been shown that both processes are not mutually exclusive and the high integration of one module can allow to promote changes across the entire module (Goswami & Polly 2010).

## Conclusion

We have shown that the evolution of the pectoral fin skeleton in batoids, the largest group of cartilaginous fishes, displays different rates on each element forming the whole structure. This is reflected by the pattern of morphological disparity through time on each skeletal element. The guitarfish-like morphology seen from the Jurassic onwards appears to be a recurrent pattern that has evolved several times in their evolution. This probably has obscured their phylogenetic relationships in past studies. Here, we demonstrate that these morphologies are the result of convergent evolution into a general shape present in most of the groups. The basal fin skeleton follows a different path than the radial portion of the fin. This includes an apparently higher phylogenetic signal carried by the skeletal anatomy, which can support previous findings of fossil phylogenetic analyses (Sansom & Wills 2013). Altogether, we found that the extreme morphologies and reduced disparity of some of the groups can be explained by a process of phenotypic integration, from which the external morphology can develop into different shapes and environments.

## Supplementary Information

Below is the link to the electronic supplementary material.

Supplementary file1 (PDF 4844 KB)

Supplementary file2 (XLSX 42 KB)

## Data Availability

Electronic supplementary material is available online at https://github.com/Faviel-LR/Batoid_Fins.

## Code availability

Scripts used to analyse the date can be found online at: https://github.com/Faviel-LR/Batoid_Fins
