# Supplementary material for: Evolution of the batoidea pectoral fin skeleton: convergence, modularity, and integration driving disparity trends
Source: Evol Ecol. 2025 Feb 17;39(1):111–34. doi: 10.1007/s10682-025-10330-x (PMC7617449; doi:10.1007/s10682-025-10330-x)
Supplement: Supplementary file 1 — Supplementary file1 (PDF 4844 KB) [file 10682_2025_10330_MOESM1_ESM.pdf]

Evolution of the Batoidea Pectoral Fin Skeleton: Convergence, Modularity and Integration Driving Disparity Trends

SUPPLEMENTARY MATERIAL

López-Romero Faviel A.,[1] Villalobos-Segura Eduardo,[2] Türtscher Julia,[2, 3] Berio Fidji,[4] Stumpf Sebastian,[2] Dearden Richard P.,[5,6] Kriwet Jürgen,[2, 3] Maldonado Ernesto[1]*.

1. EvoDevo Research Group, Unidad de Sistemas Arrecifales, Instituto de Ciencias del Mar y Limnología, Universidad Nacional Autónoma de México, Puerto Morelos, Quintana Roo, México;

2 University of Vienna, Faculty of Earth Sciences, Geography and Astronomy, Department of Palaeontology, Evolutionary Morphology Research Group, Josef-Holaubek-Platz 2, 1190, Vienna, Austria

3 University of Vienna, Vienna Doctoral School of Ecology and Evolution (VDSEE), Djerassiplatz 1, 1030, Vienna, Austria

4 Department of Zoology, Stockholm University, Svante Arrhenius väg 18B, 114 18, Stockholm, Sweden.

5 Vertebrate Evolution, Development, and Ecology, Naturalis Biodiversity Center, Darwinweg 2, Leiden, 2333 CR, The Netherlands

6 University of Birmingham, School of Geography, Earth & Environmental Sciences, University of Birmingham, Edgbaston, Birmingham B15 2TT, UK

*Correspondence to: Ernesto Maldonado.
Unidad Académica de Sistemas Arrecifales, Instituto de Ciencias del Mar y Limnología, Universidad Nacional Autónoma de México, C.P. 77580 Puerto Morelos, Quintana Roo, México.
email: ernesto@cmarl.unam.mx

Supplementary Table 1. Landmark definitions used for the present study.

Landmark/ Curve	
L1	Anterior midline of the coracoid bar
C1	Anterior curve of the coracoid bar
L2	Inner coracoid bar-propterygium joint
C2	Inner curve of the first propterygium segment
L3	Anterior inner propterygium joint with second propterygium segment
L4	Anterior outer propterygium joint with second propterygium segment
C3	Outer curve of the first propterygium segment
L5	Outer coracoid bar-propterygium joint
L6	Posterior midline of the coracoid bar
C4	Posterior curve of the coracoid bar
L7	Inner coracoid bar-metapterygium joint
C5	Inner curve of the first metapterygium segment
L8	Posterior inner metapterygium joint with second metapterygium segment
L9	Posterior outer metapterygium joint with second metapterygium segment
C6	Outer curve of the first metapterygium segment
L10	Outer coracoid bar-metapterygium joint
C7	Coracoid bar - mesopterygium joint curve

Supplementary Table 2. Morphological disparity as sum of variances for all groups. Subsets of taxonomic groups, temporal groups, habitat categories, and swimming categories. All (All landmark configurations), CB (Coracoid Bar configuration), PR (Propterygium), MT (Metapterygium). N (number of samples per group) obs (observed disparity) bs.median (bootstrapped median disparity)

	All			CB		PR		MT	
subsets	n	obs	bs.median	obs	bs.median	obs	bs.median	obs	bs.median
Rhinop	38	0.018	0.018	0.01	0.01	0.014	0.014	0.006	0.006
Myliob	131	0.02	0.02	0.039	0.039	0.024	0.023	0.006	0.006
Torped	35	0.036	0.035	0.012	0.011	0.032	0.03	0.041	0.04
Rajifo	91	0.012	0.012	0.026	0.026	0.015	0.015	0.004	0.004
Orecto	5	0.039	0.032	0.025	0.02	0.081	0.059	0.014	0.01
Pristi	6	0.019	0.015	0.012	0.011	0.05	0.044	0.021	0.018
Squati	16	0.005	0.005	0.003	0.003	0.015	0.014	0.007	0.006
Cr_Raj	3	0.004	0.002	0.017	0.012	0.026	0.025	0.005	0.004
Cr_Scl	6	0.025	0.02	0.013	0.011	0.024	0.021	0.035	0.031
Cr_Rhi	12	0.02	0.019	0.037	0.03	0.013	0.012	0.007	0.007
Eo_Myl	9	0.01	0.01	0.029	0.027	0.012	0.012	0.003	0.003
Jr_Squ	3	0.018	0.013	0.018	0.011	0.044	0.034	0.01	0.006
Jr_Bat	11	0.011	0.01	0.017	0.015	0.009	0.008	0.005	0.005
Batoids	288	0.072	0.072	0.07	0.069	0.063	0.063	0.023	0.023
Sharks	25	0.022	0.022	0.017	0.016	0.082	0.08	0.015	0.014
Cr_Bat	21	0.055	0.054	0.049	0.047	0.028	0.028	0.023	0.02
Eo_Bat	10	0.067	0.062	0.049	0.044	0.038	0.037	0.026	0.025
Jr_Sha	5	0.047	0.042	0.033	0.029	0.086	0.065	0.009	0.008
Jr_Bat	11	0.011	0.01	0.017	0.015	0.009	0.008	0.005	0.005
Deep	82	0.025	0.026	0.029	0.028	0.021	0.021	0.01	0.008
FreshW	19	0.006	0.006	0.011	0.01	0.009	0.009	0.003	0.002
Reef	41	0.073	0.07	0.088	0.084	0.056	0.055	0.038	0.036
Shelf	146	0.071	0.071	0.087	0.086	0.051	0.049	0.027	0.026
Undul	191	0.05	0.05	0.041	0.041	0.049	0.049	0.005	0.004
Oscil	36	0.008	0.008	0.022	0.021	0.012	0.012	0.009	0.009
AxUnd	61	0.059	0.058	0.018	0.018	0.034	0.034	0.051	0.049

Supplementary Table 3. Pairwise comparison of the disparity by groups for the whole landmark configuration. Upper triangle indicates the P value, lower triangle indicates the statistic W of the Wilcoxon test. Bold indicates p<0.05

	Rhinop	Myliob	Torped	Rajifo	Orecto	Pristi	Squati	Cr_Raj	Cr_Scl	Cr_Rhi	Eo_Myl	Jr_Squ	Jr_Bat
Rhinop		**>0.001**	**>0.001**	**>0.001**	**>0.001**	**0.008**	**>0.001**	**>0.001**	**0.0333**	1	**>0.001**	**>0.001**	**>0.001**
Myliob	3007		**>0.001**	**>0.001**	**>0.001**	**>0.001**	**>0.001**	**>0.001**	1	**0.0012**	**>0.001**	**>0.001**	**>0.001**
Torped	183	335		**>0.001**	1	**>0.001**	**>0.001**	**>0.001**	**>0.001**	**>0.001**	**>0.001**	**>0.001**	**>0.001**
Rajifo	9854	10000	9992		**>0.001**	**>0.001**	**>0.001**	**>0.001**	**>0.001**	**>0.001**	**>0.001**	0.1271	**>0.001**
Orecto	732	920	5938	58		**>0.001**	**>0.001**	**>0.001**	**>0.001**	**>0.001**	**>0.001**	**>0.001**	**>0.001**
Pristi	6590	7701	9862	2348	9491		**>0.001**	**>0.001**	**>0.001**	**>0.001**	**>0.001**	**0.01802**	**>0.001**
Squati	10000	10000	10000	10000	10000	9905		**>0.001**	**>0.001**	**>0.001**	**>0.001**	**>0.001**	**>0.001**
Cr_Raj	10000	10000	10000	10000	10000	10000	9770		**>0.001**	**>0.001**	**>0.001**	**>0.001**	**>0.001**
Cr_Scl	3558	4880	9424	401	8725	2550	0	0		0.169	**>0.001**	**>0.001**	**>0.001**
Cr_Rhi	4690	6764	9799	119	9264	3291	0	0	6255		**>0.001**	**>0.001**	**>0.001**
Eo_Myl	9780	9958	9979	6990	9882	8306	1246	171	9611	9795		**0.0022**	1
Jr_Squ	7927	9069	9936	3714	9647	6503	1100	1083.5	8429	8138	3292		**>0.001**
Jr_Bat	9965	10000	10000	8552	9994	8693	20	0	9839	9977	4938	6450	

	Batoid	Sharks	Cr_Bat	Eo_Bat	Jr_Sha	Jr_Bat
Batoids		**>0.001**	**>0.001**	**>0.001**	**>0.001**	**>0.001**
Sharks	10000		**>0.001**	**0.0022**	**>0.001**	**>0.001**
Cr_Bat	9683	39		0.11734	**>0.001**	**>0.001**
Eo_Bat	7194	3448	3911		**>0.001**	**>0.001**
Jr_Sha	9990	1973	7384	6541		**>0.001**
Jr_Bat	10000	9989	10000	8376	9864	

	Deep	Fresh W	Reef	Shelf
Deep		**>0.001**	**>0.001**	**>0.001**
FreshW	10000		**>0.001**	**>0.001**
Reef	1	0		1
Shelf	0	0	4505	

	Undul	Oscil	AxUnd
Undul		**>0.001**	**>0.001**
Oscil	10000		**>0.001**
AxUnd	52	0	

Supplementary Table 4. Pairwise comparison of the disparity by groups for the coracoid bar landmark configuration. Upper triangle indicates the P value, lower triangle indicates the statistic W of the Wilcoxon test. Bold indicates p<0.05

	Rhinop	Myliob	Torped	Rajifo	Orecto	Pristi	Squati	Cr_Raj	Cr_Scl	Cr_Rhi	Eo_Myl	Jr_Squ	Jr_Bat
Rhinop		**>0.001**	**>0.001**	**>0.001**	**>0.001**	1	**>0.001**	1	1	**>0.001**	**>0.001**	**>0.001**	**>0.001**
Myliob	0		**>0.001**	**>0.001**	**>0.001**	**>0.001**	**>0.001**	**>0.001**	**>0.001**	0.0761	**>0.001**	**>0.001**	**>0.001**
Torped	2939	10000		**>0.001**	**>0.001**	1	**>0.001**	1	0.1148	**>0.001**	**>0.001**	1	**>0.001**
Rajifo	0	9999	0		**>0.001**	**>0.001**	**>0.001**	**>0.001**	**>0.001**	0.646	1	**>0.001**	**>0.001**
Orecto	685	10000	998	9215		**>0.001**	**>0.001**	**>0.001**	**>0.001**	**>0.001**	**>0.001**	**>0.001**	**>0.001**
Pristi	4323	10000	5842	10000	9125		**>0.001**	0.4779	1	**>0.001**	**>0.001**	0.265	**>0.001**
Squati	10000	10000	10000	10000	10000	10000		**>0.001**	**>0.001**	**>0.001**	**>0.001**	**>0.001**	**>0.001**
Cr_Raj	4080	10000	4736	10000	8692	3882	600		0.2168	**>0.001**	**>0.001**	1	**>0.001**
Cr_Scl	4885	10000	6302	10000	9240	5286	50	6220		**>0.001**	**>0.001**	**0.0057**	**>0.001**
Cr_Rhi	589	6350	921	3919	2674	814	0	972	678		1	**>0.001**	**>0.001**
Eo_Myl	0	9774	34	4145	1438	57	0	174	28	5810		**>0.001**	**>0.001**
Jr_Squ	2898	10000	4151	10000	8537	3805	900	4777	3382	8855	9792		**>0.001**
Jr_Bat	202	10000	1178	10000	7905	1259	0	3104	979	8410	9747	2945	

	Batoids	Sharks	Cr_Bat	Eo_Bat	Jr_Sha	Jr_Bat
Batoids		**>0.001**	**>0.001**	**>0.001**	**>0.001**	**>0.001**
Sharks	10000		**>0.001**	**>0.001**	**>0.001**	**0.0078**
Cr_Bat	9908	10		1	**>0.001**	**>0.001**
Eo_Bat	8901	150	5669		**>0.001**	**>0.001**
Jr_Sha	10000	3080	8989	7594		**>0.001**
Jr_Bat	10000	6420	9999	9923	6990	

	Deep	FreshW	Reef	Shelf
Deep		**>0.001**	**>0.001**	**>0.001**
FreshW	10000		**>0.001**	**>0.001**
Reef	0	0		5.10E-02
Shelf	0	0	3924	

	Undul	Oscil	AxUnd
Undul		**>0.001**	**>0.001**
Oscil	10000		**>0.001**
AxUnd	10000	8939	

Supplementary Table 5. Pairwise comparison of the disparity by groups for the propterygium landmark configuration. Upper triangle indicates the P value, lower triangle indicates the statistic W of the Wilcoxon test. Bold indicates p<0.05

	Rhinop	Myliob	Torped	Rajifo	Orecto	Pristi	Squati	Cr_Raj	Cr_Scl	Cr_Rhi	Eo_Myl	Jr_Squ	Jr_Bat
Rhinop		**>0.001**	**>0.001**	**>0.001**	**>0.001**	**>0.001**	1	**0.0185**	**>0.001**	**>0.001**	**>0.001**	**>0.001**	**>0.001**
Myliob	1		**>0.001**	**>0.001**	**>0.001**	**>0.001**	**>0.001**	1	**>0.001**	**>0.001**	**>0.001**	**>0.001**	**>0.001**
Torped	3	1844		**>0.001**	**>0.001**	0.1451	**>0.001**	**>0.001**	**>0.001**	**>0.001**	**>0.001**	0.0531	**>0.001**
Rajifo	2391	9976	9973		**>0.001**	**>0.001**	**>0.001**	**0.0185**	**>0.001**	**>0.001**	**>0.001**	**>0.001**	**>0.001**
Orecto	127	309	605	185		**>0.001**	**>0.001**	**>0.001**	**>0.001**	**>0.001**	**>0.001**	**>0.001**	**>0.001**
Pristi	161	3052	3726	390	7692		**>0.001**	**>0.001**	**>0.001**	**>0.001**	**>0.001**	**0.0064**	**>0.001**
Squati	5111	9986	9985	7550	9876	9793		**0.0185**	**>0.001**	**>0.001**	**>0.001**	**>0.001**	**>0.001**
Cr_Raj	3500	5980	8659	3500	9805	7601	3500		1	**0.0185**	**0.0185**	**>0.001**	**0.0185**
Cr_Scl	1628	7022	8926	2244	9754	7976	1591	5500		**>0.001**	**>0.001**	**>0.001**	**>0.001**
Cr_Rhi	7563	10000	10000	9045	9936	9938	7259	6500	9032		1	**>0.001**	**>0.001**
Eo_Myl	7863	10000	10000	9214	9947	9947	7609	6500	9143	5611		**>0.001**	**>0.001**
Jr_Squ	2900	2924	3614	2900	8774	6606	2831	2068	2877	2739	2440		**>0.001**
Jr_Bat	9964	10000	10000	10000	10000	10000	9752	6500	9853	9085	8076	8365	

	Batoids	Sharks	Cr_Bat	Eo_Bat	Jr_Sha	Jr_Bat
Batoids		**>0.001**	**>0.001**	**>0.001**	1	**>0.001**
Sharks	2069		**>0.001**	**>0.001**	**0.0031**	**>0.001**
Cr_Bat	10000	9949		**0.031**	**>0.001**	**>0.001**
Eo_Bat	9073	9320	3739		**>0.001**	**>0.001**
Jr_Sha	4275	6516	459	1547		**>0.001**
Jr_Bat	10000	10000	10000	9401	10000	

	Deep	Fresh W	Reef	Shelf
Deep		**>0.001**	**>0.001**	**>0.001**
FreshW	10000		**>0.001**	**>0.001**
Reef	0	0		**>0.001**
Shelf	0	0	7460	

	Undul	Oscil	AxUnd
Undul		**>0.001**	**>0.001**
Oscil	10000		**>0.001**
AxUnd	10000	0	

Supplementary Table 6. Pairwise comparison of the disparity by groups for the metapterygium landmark configuration. Upper triangle indicates the P value, lower triangle indicates the statistic W of the Wilcoxon test. Bold indicates p<0.05

	Rhinop	Myliob	Torped	Rajifo	Orecto	Pristi	Squati	Cr_Raj	Cr_Scl	Cr_Rhi	Eo_Myl	Jr_Squ	Jr_Bat
Rhinop		1	**>0.001**	**>0.001**	**>0.001**	**>0.001**	1	**>0.001**	**>0.001**	**>0.001**	**>0.001**	1	**0.0035**
Myliob	4985		**>0.001**	**>0.001**	**>0.001**	**>0.001**	0.9532	**>0.001**	**>0.001**	**>0.001**	**>0.001**	1	**>0.001**
Torped	0	0		**>0.001**	**>0.001**	**>0.001**	**>0.001**	**>0.001**	**>0.001**	**>0.001**	**>0.001**	**>0.001**	**>0.001**
Rajifo	9847	10000	10000		**>0.001**	**>0.001**	**>0.001**	**0.0084**	**>0.001**	**>0.001**	**>0.001**	**>0.001**	**>0.001**
Orecto	1811	1852	10000	257		**>0.001**	**>0.001**	**>0.001**	**>0.001**	**>0.001**	**>0.001**	**>0.001**	**>0.001**
Pristi	462	413	9988	300	2485		**>0.001**	**>0.001**	**>0.001**	**>0.001**	**>0.001**	**>0.001**	**>0.001**
Squati	4124	3974	10000	201	7930	9438		**>0.001**	**>0.001**	0.7406	**>0.001**	1	**>0.001**
Cr_Raj	8758	9514	10000	3420	9144	9802	8868		**>0.001**	**>0.001**	**0.00689**	**>0.001**	**>0.001**
Cr_Scl	333	345	7916	0	561	1477	396	76		**>0.001**	**>0.001**	**>0.001**	**>0.001**
Cr_Rhi	2982	2683	10000	218	7621	9325	3938	564	9535		**>0.001**	1	**>0.001**
Eo_Myl	9990	10000	10000	9368	9889	9708	9988	6600	10000	9979		**>0.001**	**>0.001**
Jr_Squ	4612	4928	10000	1228	7624	9185	4792	2404	9672	5105	1200		0.09
Jr_Bat	6668	6888	10000	1033	8647	9621	7264	2652	9784	8112	255	6320	

	Batoid	Sharks	Cr_Bat	Eo_Bat	Jr_Sha	Jr_Bat
Batoids		**>0.001**	**0.0064**	1	**>0.001**	**>0.001**
Sharks	9903		**>0.001**	0.2189	**>0.001**	**>0.001**
Cr_Bat	6442	2232		1	**>0.001**	**>0.001**
Eo_Bat	4598	4000	4853		0.0735	0.15
Jr_Sha	10000	9875	9663	6152		**>0.001**
Jr_Bat	10000	10000	9971	6055	7599	

	Deep	FreshW	Reef	Shelf
Deep		**>0.001**	**>0.001**	**>0.001**
FreshW	9976		**>0.001**	**>0.001**
Reef	1	0		**>0.001**
Shelf	18	0	9003	

	Undul	Oscil	AxUnd
Undul		**>0.001**	**>0.001**
Oscil	0		**>0.001**
AxUnd	0	0	

Supplementary Table 7. Evolutionary rates and shift by clades, p value indicates the positive (p>0.975) or negative (p<0.025) shifts from the search.shift function in RRphylo. All the configurations and isolated elements comparisons. * Result found with the “auto” parameter in search.shift.

	Node	Group	rate.difference	p.value
Full configuration		All	0.001665675	0.915
	406	Sela	0.0002815367	0.563
	213	Bats	0.0017804218	0.94
	403	Jbat	0.0003566254	0.892
	401	Scle	0.0014195306	**0.9995**
	216	Rhin	0.002341807	0.948
	384	Torp	0.0006783165	0.891
	322	Raji	0.0027842153	**0.999**
	249	Mylio	0.001021813	0.791
	249	Mylio*	-0.0009675225	**0.001**
Coracoid bar		All	0.003401622	0.995
	406	Sela	0.0004246853	0.774
	213	Bats	0.0036484149	**0.999**
	403	Jbat	0.0007212336	**1**
	401	Scle	0.0014043769	**0.9995**
	216	Rhin	0.0055538655	**1**
	384	Torp	0.0004796465	0.946
	322	Raji	0.003950205	**1**
	249	Mylio	0.0036225049	**0.999**
Propterygium		All	0.002695441	0.981
	406	Sela	0.0005301853	0.719
	213	Bats	0.0028749438	**0.987**
	403	Jbat	0.0002726882	0.806
	401	Scle	0.0022005471	**0.9865**
	216	Rhin	0.002035749	**0.991**
	384	Torp	0.0022996407	**0.989**
	322	Raji	0.0037849555	**1**
	249	Mylio	0.0028783547	0.984
Metapterygium		All	0.001661485	0.997
	406	Sela	0.0004578208	0.922
	213	Bats	0.0017612708	**0.995**
	403	Jbat	0.0007751719	**0.998**
	401	Scle	0.0020142031	**1**
	216	Rhin	0.0021474323	**1**
	384	Torp	0.0024454247	**1**
	322	Raji	0.0014385677	**0.99**
	249	Mylio	0.0018033827	**1**

Supplementary table 8. Results from the overFit function to account for phylogenetic uncertainty. 100 random trees were used. p.shift+ or p.shift- indicates the percentage of times the same shift was found in the random sample. And tested.trees the percentage of trees used. Bold indicates the same result as the one obtained with a single tree.

		p.shift+	p.shift-	tested.trees
Full configuration	all.cla	0	0	1
	406	0	0	1
	213	0	0	1
	403	0	0	1
	401	**1**	0	1
	216	0	0	1
	384	0	0	1
	322	**1**	0	1
	249	0	0	1
Coracoid bar	all.cla	1	0	1
	406	0	0	1
	213	**1**	0	1
	403	**1**	0	1
	401	**1**	0	1
	216	**0.99**	0	1
	384	0.13	0	1
	322	**1**	0	1
	249	**1**	0	1
Propterygium	all.cla	0.17	0	1
	406	0	0	1
	213	**0.42**	0	1
	403	0	0	1
	401	**1**	0	1
	216	0.12	0	1
	384	**0.86**	0	1
	322	**1**	0	1
	249	0.18	0	1
Metapterygium	all.clades	0.96	0	1
	406	0	0	1
	213	**0.98**	0	1
	403	**1**	0	1
	401	**1**	0	1
	216	**0.99**	0	1
	384	**1**	0	1
	322	0.79	0	1
	249	**0.98**	0	1

Supplementary Table 9. Evolutionary rates as sigma^2 from the Extant set comparing habitat groups and swimming categories for the whole configuration (All), coracoid bar (CB), propterygium (PR) or metapterygium (MT).

	All	CB	PR	MT
Deep	1.41E-05	2.88E-05	1.90E-05	1.27E-06
Freshwater	2.08E-05	6.86E-05	4.97E-05	1.61E-06
Reef	7.68E-05	8.41E-05	7.13E-05	2.20E-05
Shelf	3.41E-05	5.97E-05	2.88E-05	1.59E-05
AxUnd	6.11E-05	9.37E-06	3.40E-05	3.74E-05
Oscil	1.92E-05	3.22E-04	4.80E-05	1.21E-05
Undul	2.82E-05	8.09E-05	3.48E-05	2.61E-06

Supplementary Table 10. Results from the manova.gls test for the shape variables and the groups. Bold indicates a significant interaction at $p<0.05$

Structure	Group	Test stat	Pr(>Stat)
All	Amb	0.1245	0.29
	Mov	0.01807	0.738
CB	Amb	0.1715	**0.001**
	Mov	0.4153	**0.001**
PR	Amb	0.08498	**0.041**
	Mov	0.1966	**0.007**
MT	Amb	0.09454	0.26
	Mov	0.109	**0.041**

Supplementary Table 11. Effect size pairwise comparison between the modularity hypotheses tested. The CR value is indicated for each configuration. Lower triangle indicates the effect size, and upper triangle the p value. Bold indicates significant (p<0.05)

Sharks and Batoids

	No_Modules	MT.AL	MT.GP	MT.GM	MT.PM
CR		0.8466	0.8673	0.9011	0.8762
No_Md	0	**6.80E-17**	**2.57E-11**	**3.08E-09**	**6.86E-14**
MT.AL	8.350497	0	5.71E-01	1.13E-01	2.37E-01
MT.GP	6.669175	0.5667709	0	3.52E-01	6.07E-01
MT.GM	5.9274	1.5832396	0.9300734	0	6.21E-01
MT.PM	7.490543	1.1830142	0.5137082	0.4941889	0

Batoids

CR		0.852	0.8729	0.9057	0.8779
No_Md	0	**1.66E-17**	**1.42E-11**	**2.57E-09**	**1.46E-14**
MT.AL	8.515617	0	5.28E-01	9.22E-02	2.68E-01
MT.GP	6.756021	0.6310762	0	3.35E-01	7.02E-01
MT.GM	5.957238	1.6838353	0.9638557	0	5.07E-01
MT.PM	7.691361	1.1081814	0.3823374	0.6640423	0

Supplementary Figures

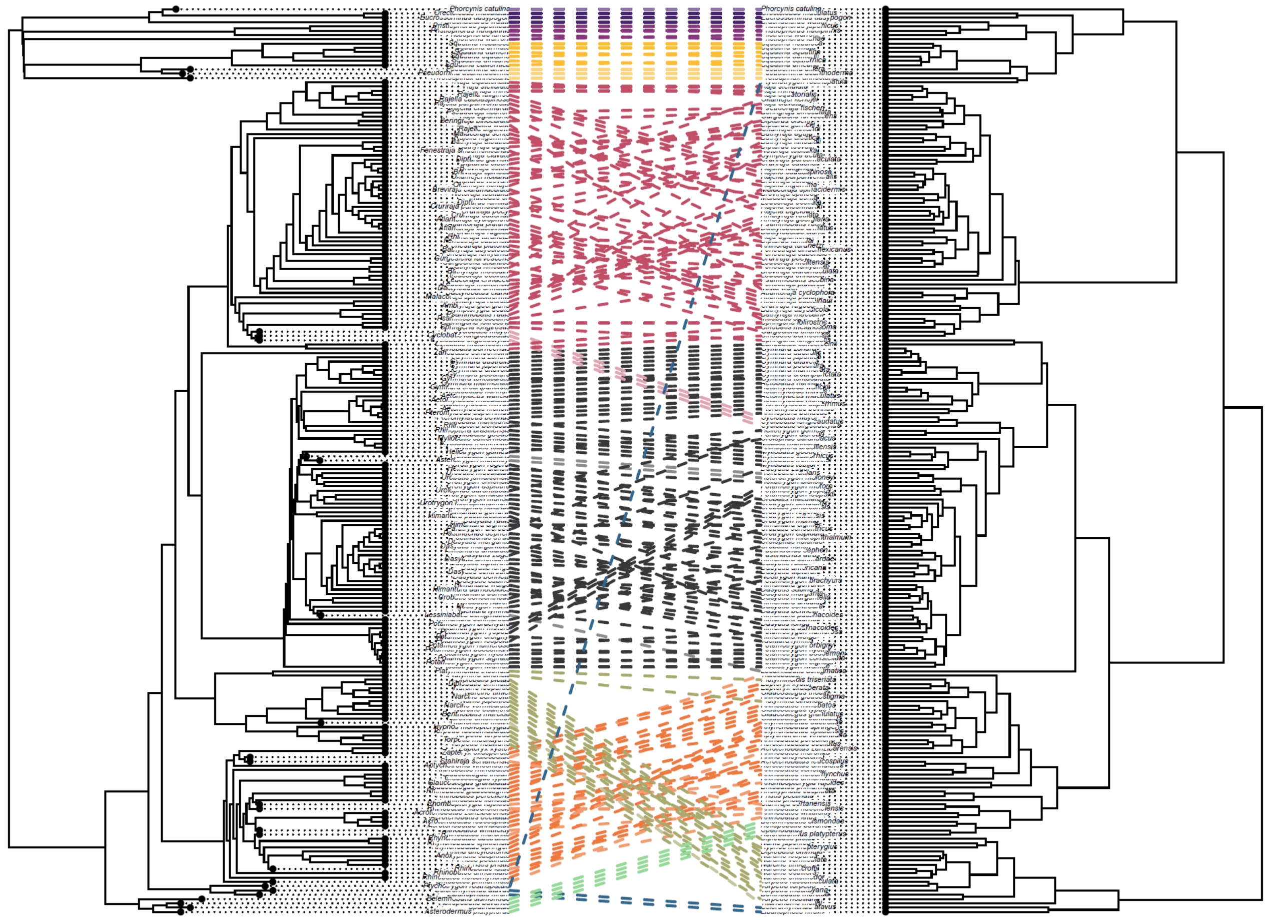

Supplementary Figure 1. Phylogenetic tree (left) and distance-based tree (right) using the pairwise procrustes distances matrix followed by a UPGMA clustering. Lines indicate the taxonomic group.

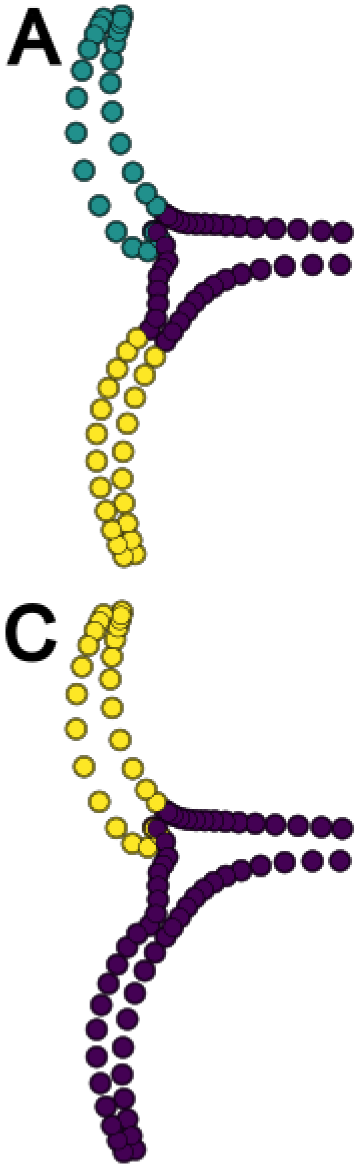

Supplementary figure 2. Tested modularity hypotheses. A) Three modules with each element separated. B) Two modules with propterygium and coracoid bar as a single module. C) Two modules as the metapterygium and coracoid bar as a single module. D) Two modules with the propterygium and metapterygium as a single module.

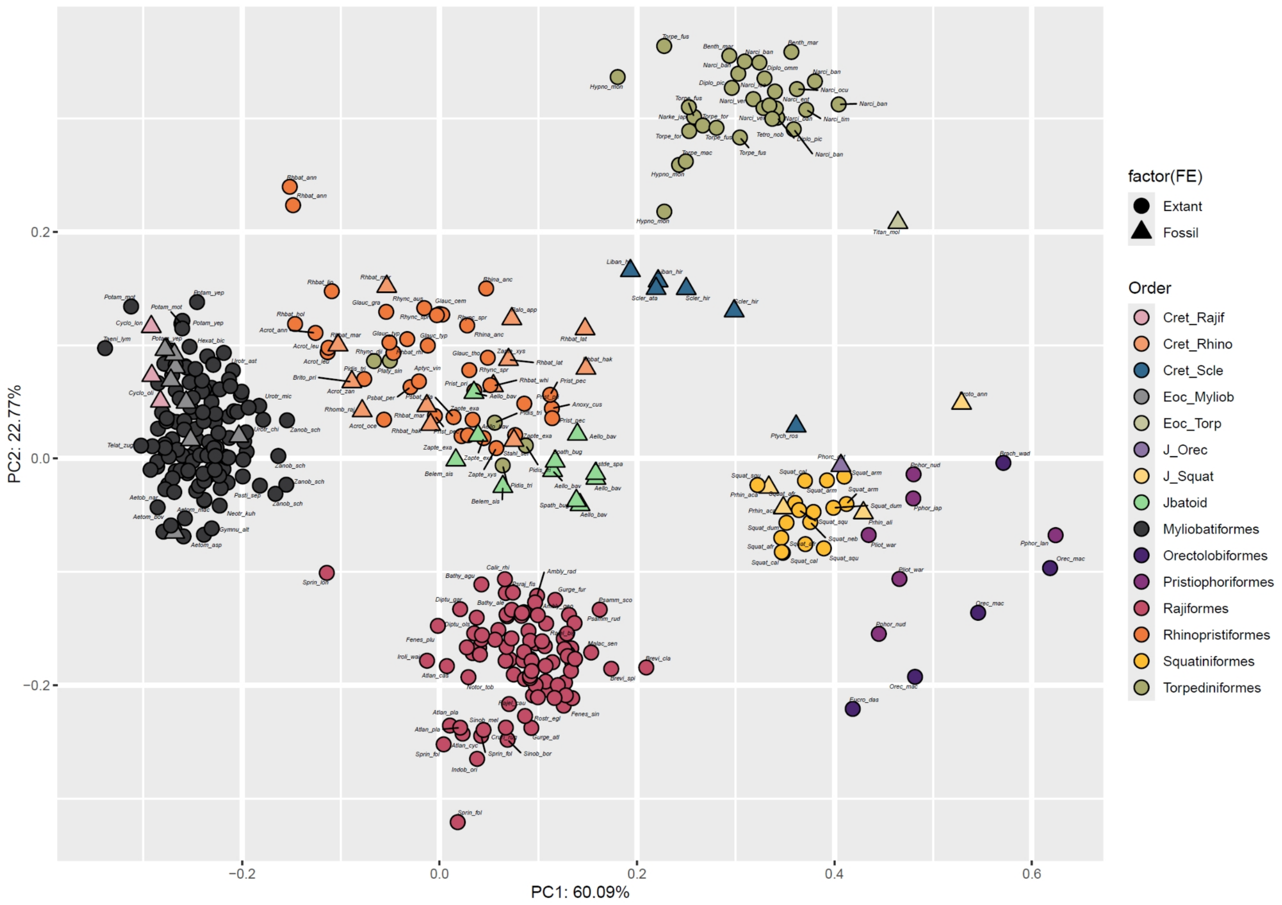

Supplementary Figure 3. Principal component analysis with all the individuals used in the study considering the full landmark configuration. Colour code indicate the taxonomic group.

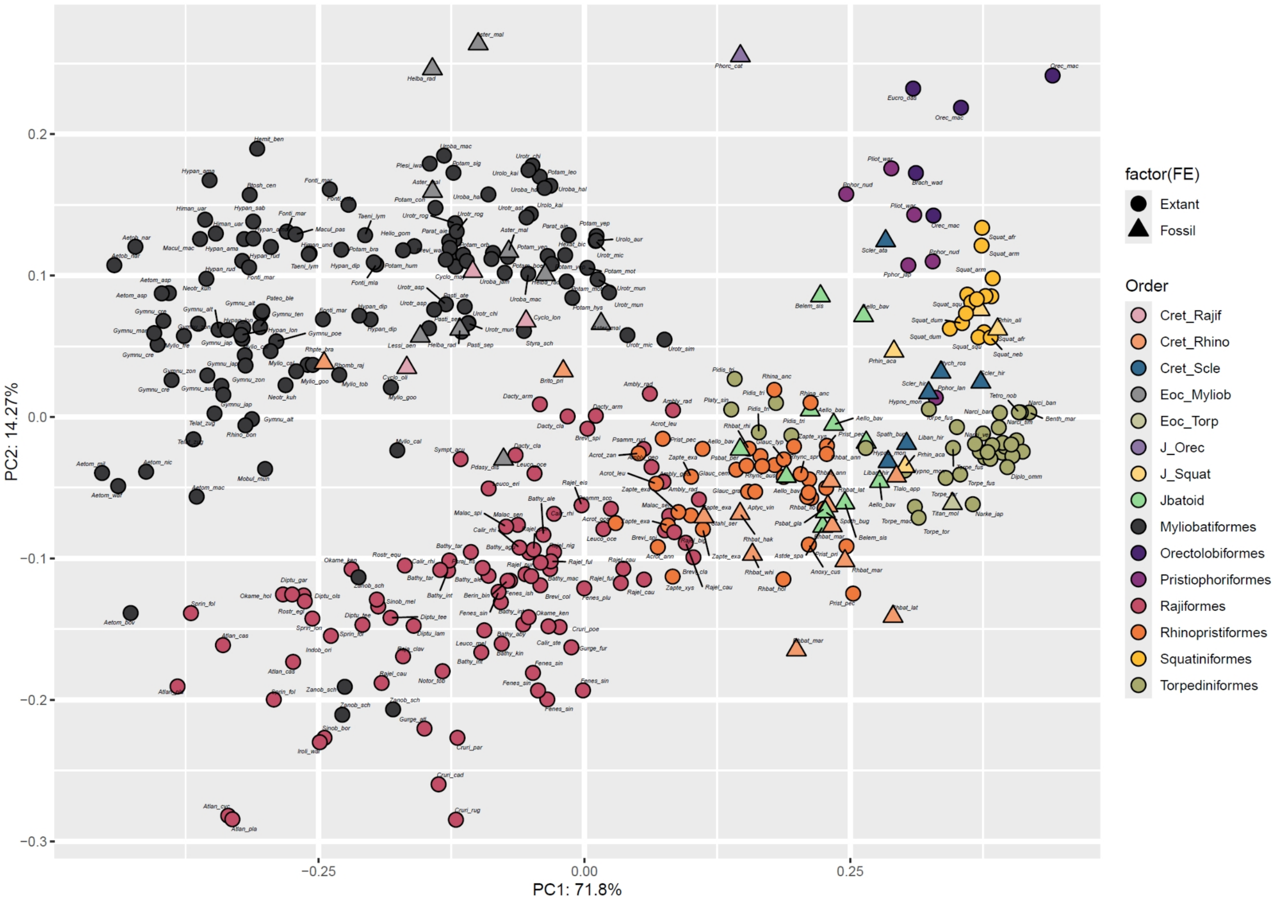

Supplementary figure 4. Principal component analysis of all the individuals with only the coracoid bar landmark configuration. Colour code indicate the taxonomic group.

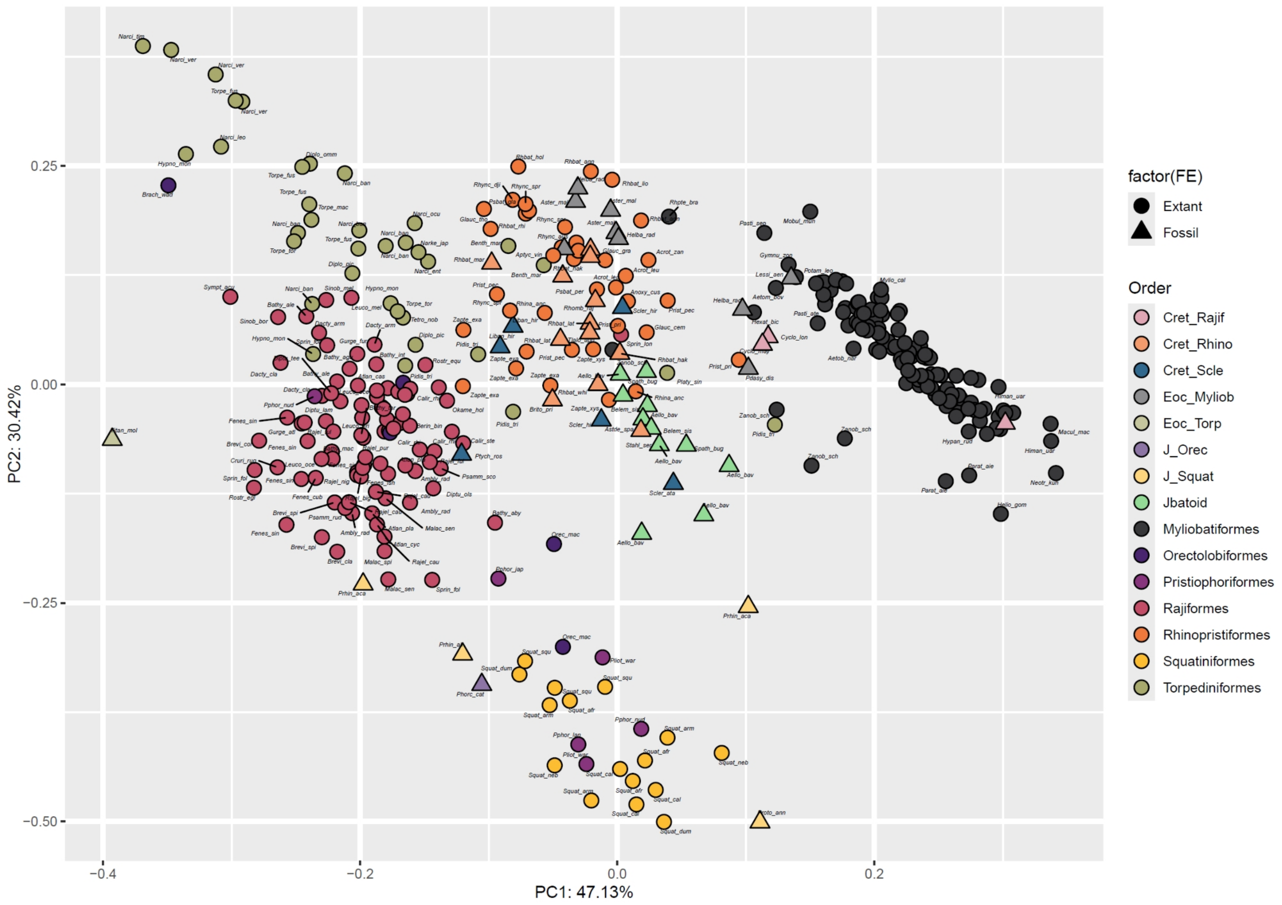

Supplementary figure 5. Principal component analysis with all the individuals for the propterygium landmark configuration. Colour code indicates the taxonomic group.

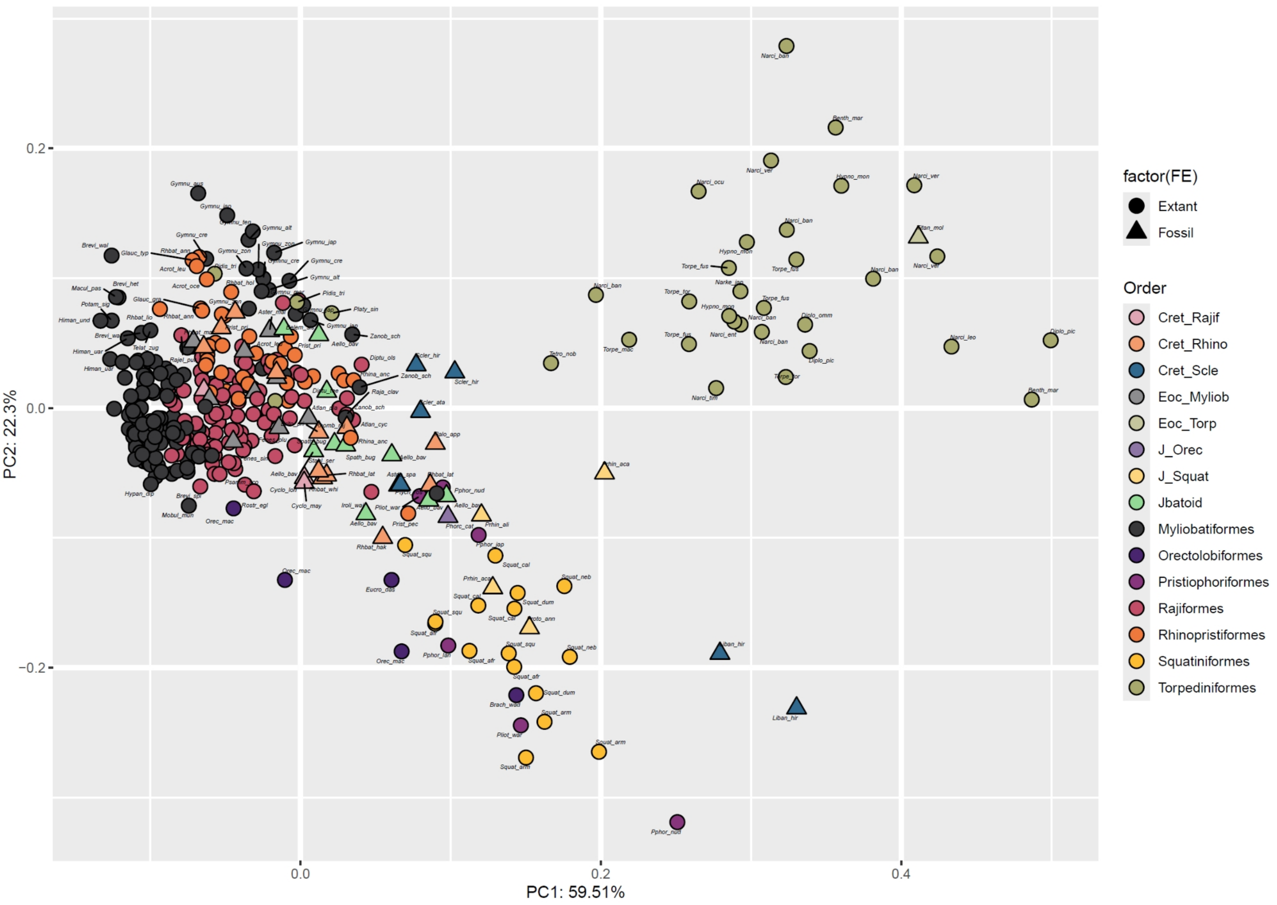

Supplementary figure 6. Principal component analysis with all the individuals for the metapterygium landmark configuration. Colour code indicates the taxonomic group.

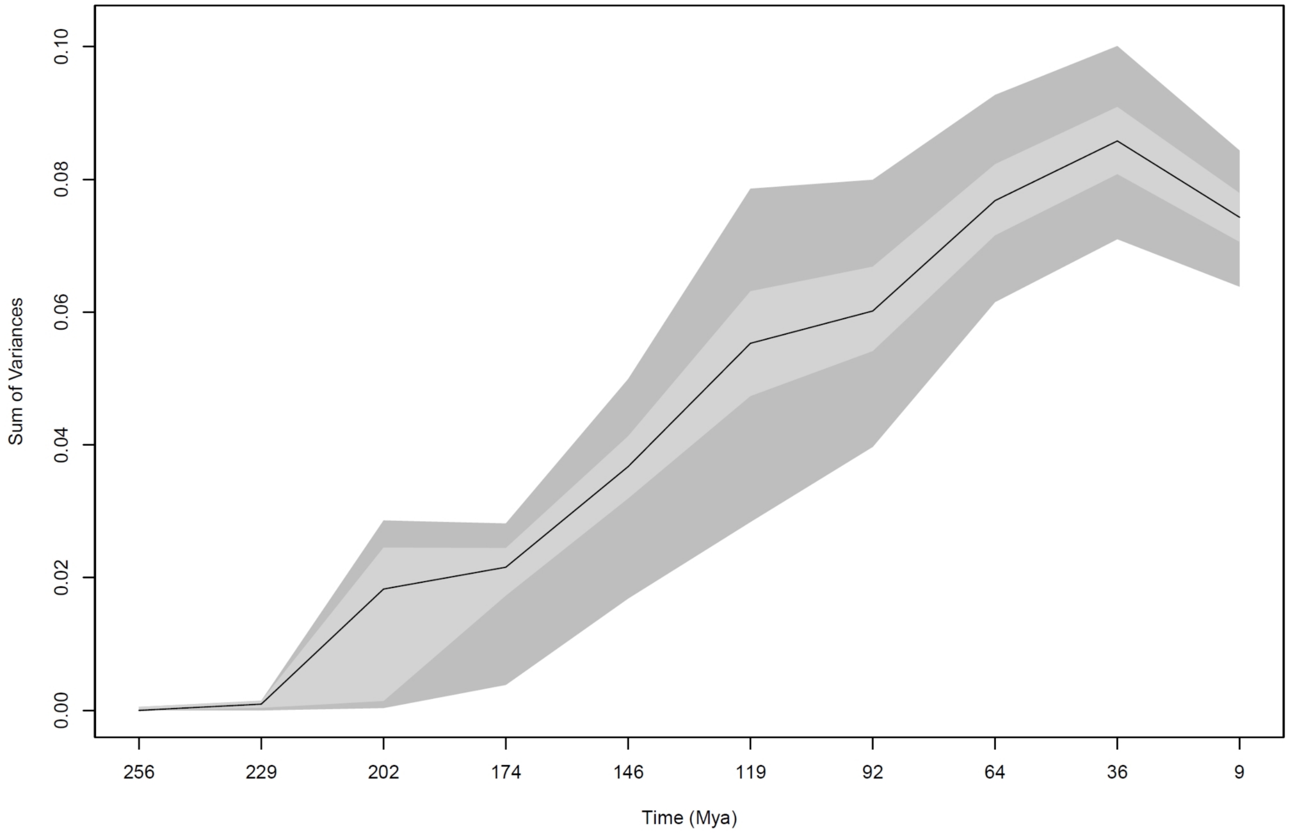

Supplementary figure 7. Disparity through time for all the individuals, including sharks and batoids. Disparity estimated as sum of variances.

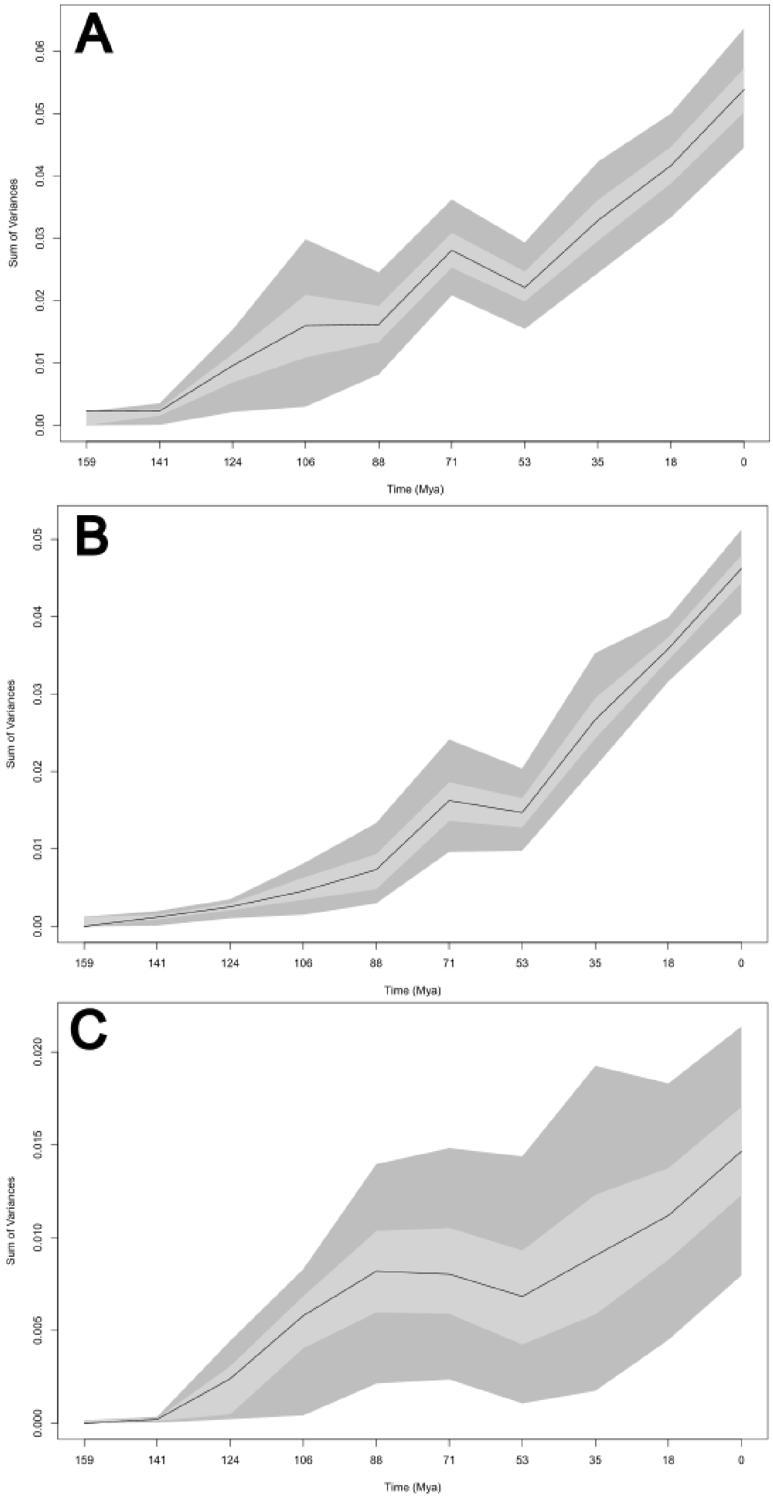

Supplementary Figure 8. Disparity through time for only batoid species. A) Coracoid bar landmark configuration. B) Propterygium landmark configuration. C) Metapterygium landmark configuration.

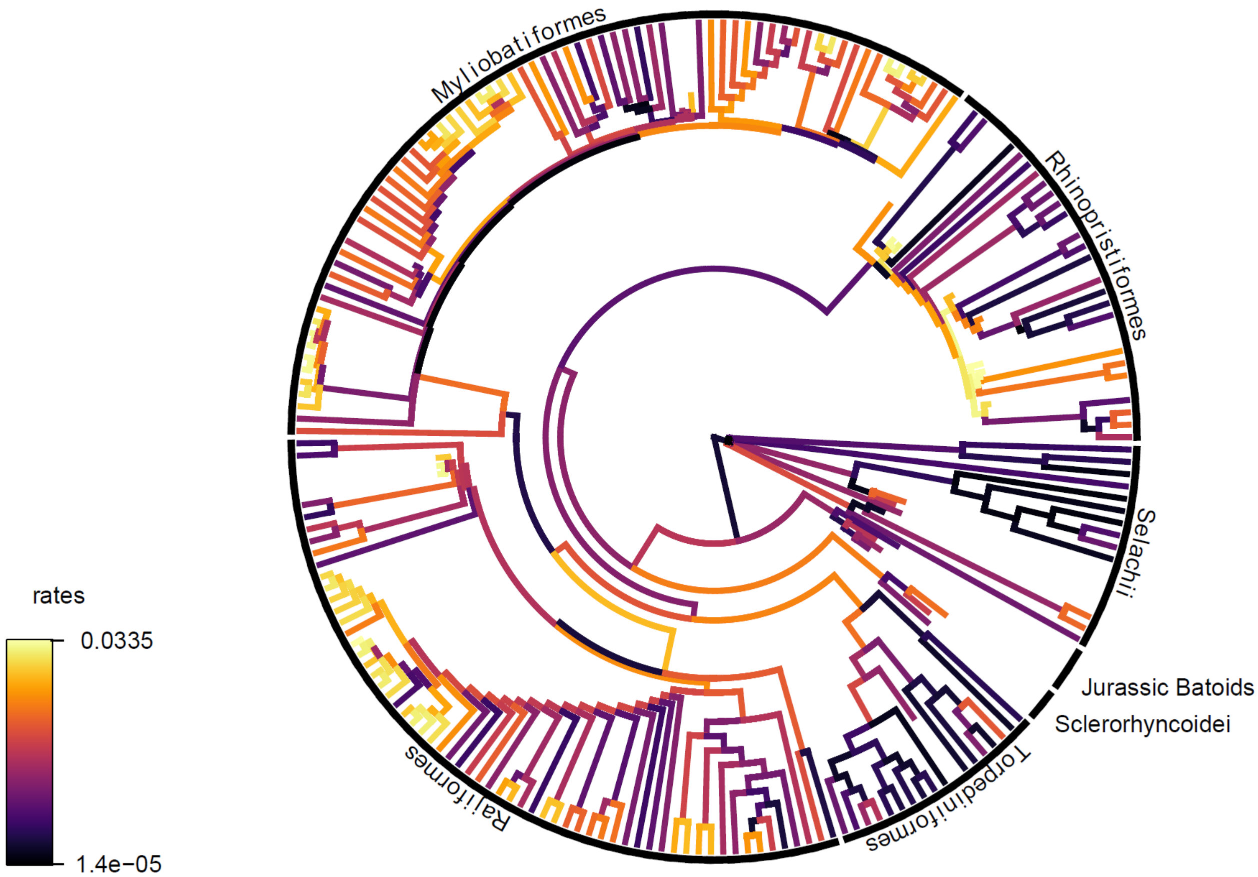

Supplementary figure 9. Evolutionary rates per branch for the coracoid bar configuration. Warmer colours indicate higher rates.

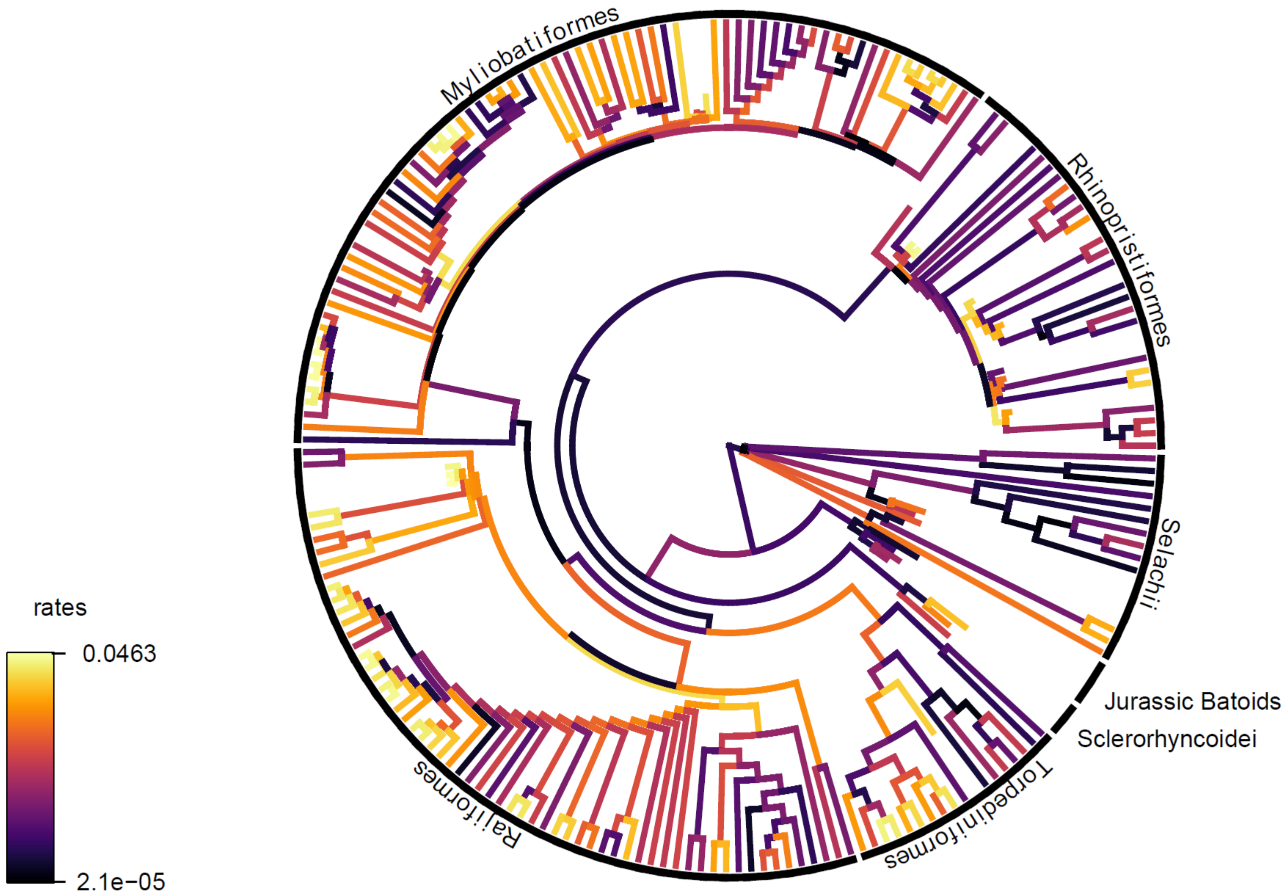

Supplementary figure 10. Evolutionary rates per branch for the propterygium configuration. Warmer colours indicate higher rates.

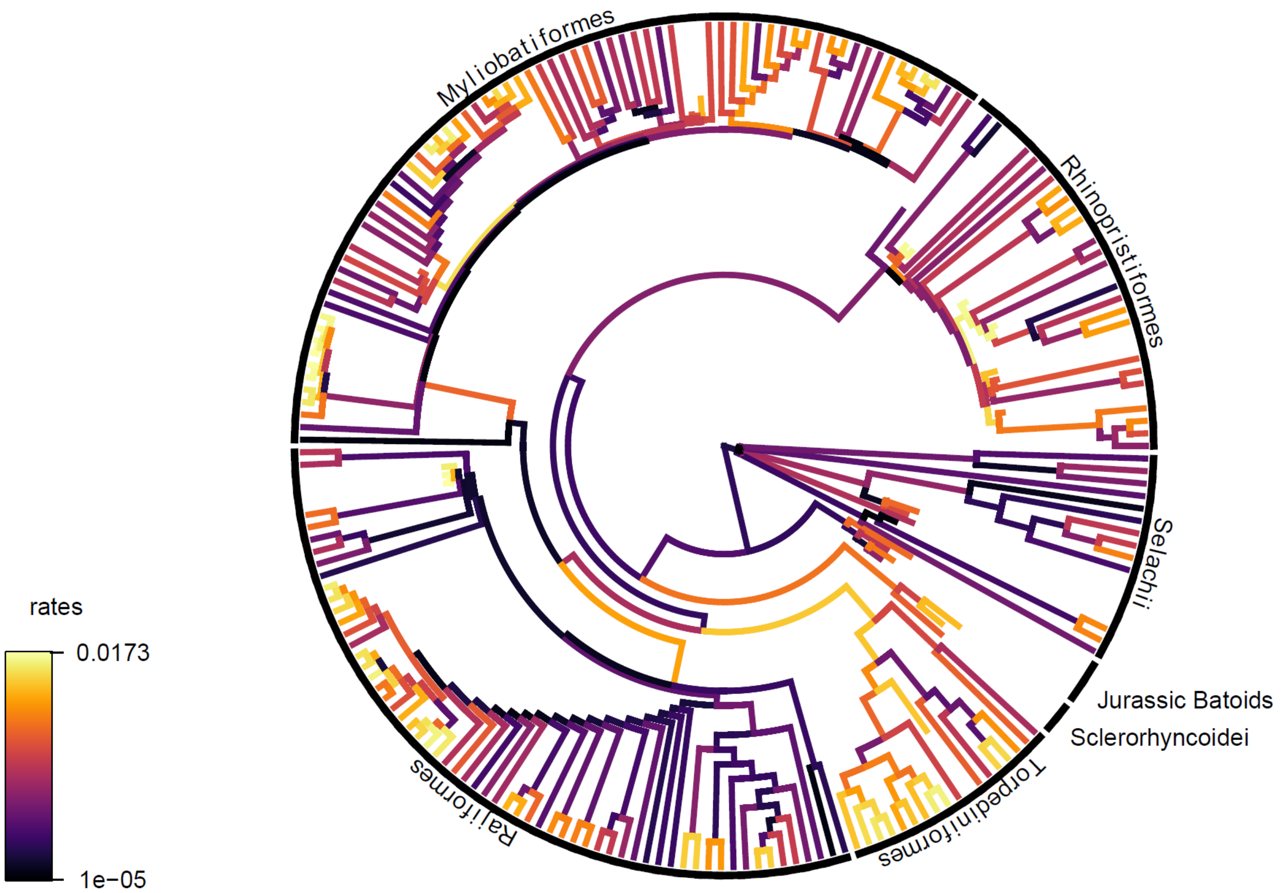

Supplementary figure 11. Evolutionary rates per branch for the propterygium configuration. Warmer colours indicate higher rates.
